# Tspan8 is expressed in breast cancer and regulates E‐cadherin/catenin signalling and metastasis accompanied by increased circulating extracellular vesicles

**DOI:** 10.1002/path.5281

**Published:** 2019-06-18

**Authors:** Maren Voglstaetter, Andreas R Thomsen, Jerome Nouvel, Arend Koch, Paul Jank, Elena Grueso Navarro, Tanja Gainey‐Schleicher, Richa Khanduri, Andrea Groß, Florian Rossner, Carina Blaue, Clemens M Franz, Marina Veil, Gerhard Puetz, Andreas Hippe, Jochen Dindorf, Jubin Kashef, Wilko Thiele, Bernhard Homey, Celine Greco, Claude Boucheix, Andreas Baur, Thalia Erbes, Cornelius F Waller, Marie Follo, Ghamartaj Hossein, Christine Sers, Jonathan Sleeman, Irina Nazarenko

**Affiliations:** ^1^ Institute for Infection Prevention and Hospital Epidemiology; Medical Center ‐ University of Freiburg Faculty of Medicine, University of Freiburg Freiburg Germany; ^2^ Department of Radiation Oncology Medical Center, University of Freiburg Freiburg im Breisgau Germany; ^3^ German Cancer Consortium (DKTK) Partner Site Freiburg and German Cancer Research Center (DKFZ) Heidelberg Germany; ^4^ Institute of Toxicology and Genetics, Karlsruhe Institute of Technology Karlsruhe Germany; ^5^ Institute of Neuropathology, Charité Universitätsmedizin Berlin Berlin Germany; ^6^ Institute of Pathology, Charité Universitätsmedizin Berlin Berlin Germany; ^7^ DFG‐Center for Functional Nanostructures Karlsruhe Institute of Technology Karlsruhe Germany; ^8^ Institute of Clinical Chemistry and Laboratory Medicine University of Freiburg Freiburg im Breisgau Germany; ^9^ Department of Dermatology, Medical Faculty University of Düsseldorf Düsseldorf Germany; ^10^ Department of Dermatology University Hospital Erlangen Erlangen Germany; ^11^ Translational Research Center Friedrich‐Alexander‐University of Erlangen‐Nuernberg Erlangen Germany; ^12^ Institute for Photon Science and Synchrotron Radiation, Karlsruhe Institute of Technology (KIT) Eggenstein‐Leopoldshafen Germany; ^13^ Medical Faculty University of Heidelberg Mannheim Germany; ^14^ UMR‐S935, Inserm Université Paris Sud, Université Paris Saclay Villejuif France; ^15^ Department of Pain Management and Palliative Care Necker Hospital Paris France; ^16^ Department of Gynecology and Obstetrics, Faculty of Medicine University of Freiburg Freiburg Germany; ^17^ Department of Medicine I, Medical Center University of Freiburg Freiburg Germany; ^18^ Faculty of Medicine University of Freiburg Freiburg Germany; ^19^ Department of Animal Physiology, Laboratory of Developmental Biology University of Tehran Tehran Iran

**Keywords:** breast cancer, Tspan8, mesenchymal–epithelial transition, beta‐catenin signalling pathway, tetraspanins, extracellular vesicles, metastases, three‐dimensional cell culture

## Abstract

Tspan8 exhibits a functional role in many cancer types including pancreatic, colorectal, oesophagus carcinoma, and melanoma. We present a first study on the expression and function of Tspan8 in breast cancer. Tspan8 protein was present in the majority of human primary breast cancer lesions and metastases in the brain, bone, lung, and liver. In a syngeneic rat breast cancer model, Tspan8^+^ tumours formed multiple liver and spleen metastases, while Tspan8^−^ tumours exhibited a significantly diminished ability to metastasise, indicating a role of Tspan8 in metastases. Addressing the underlying molecular mechanisms, we discovered that Tspan8 can mediate up‐regulation of E‐cadherin and down‐regulation of Twist, p120‐catenin, and β‐catenin target genes accompanied by the change of cell phenotype, resembling the mesenchymal–epithelial transition. Furthermore, Tspan8^+^ cells exhibited enhanced cell–cell adhesion, diminished motility, and decreased sensitivity to irradiation. As a regulator of the content and function of extracellular vesicles (EVs), Tspan8 mediated a several‐fold increase in EV number in cell culture and the circulation of tumour‐bearing animals. We observed increased protein levels of E‐cadherin and p120‐catenin in these EVs; furthermore, Tspan8 and p120‐catenin were co‐immunoprecipitated, indicating that they may interact with each other. Altogether, our findings show the presence of Tspan8 in breast cancer primary lesion and metastases and indicate its role as a regulator of cell behaviour and EV release in breast cancer. © 2019 The Authors. *The Journal of Pathology* published by John Wiley & Sons Ltd on behalf of Pathological Society of Great Britain and Ireland.

## Introduction

The need to enhance understanding of how metastases develop and progress has initiated many studies. The role of epithelial–mesenchymal transition (EMT) has come to the fore, suggesting that a proportion of primary tumour cells acquire mesenchymal properties to pass the basal membrane, disseminate into the blood stream or lymphatic system, and colonise distant organs 1. Based on this clinical observation, it has also been proposed that a reverse process, so‐called mesenchymal–epithelial transition (MET), which triggers cancer cell redifferentiation, takes place in a portion of metastases and supports their growth 2. Despite controversial discussions and recent research showing that EMT is not important for metastases but does play a role in conferring chemoresistance in lung 3 and pancreatic cancer 4, a plethora of data support the occurrence of EMT–MET‐like processes in breast cancer 5. Whereas primary breast carcinomas, e.g. invasive ductal carcinomas, express mesenchymal markers, their metastases frequently exhibit epithelial features 6. However, the underlying mechanisms are not yet understood. In this work, we propose a new function of Tspan8 as an EMT–MET regulator in breast cancer.

Tetraspanins are transmembrane proteins that act as membrane scaffolds contributing to the arrangement of proteins in the cell membrane by organising so‐called tetraspanin‐enriched microdomains (tetraspanin web) 7. Tetraspanin Tspan8 function is linked to the regulation of cell motility, and is relevant for cancer progression. Up‐regulation of Tspan8‐coding mRNA has been detected in colorectal, pancreatic 8, gastric 9, and hepatocellular 10 carcinomas, as well as in melanoma 11 and glioma 12. It is likely that Tspan8 interacts with different proteins in different tumour types. Thus, in pancreatic cancer, CD9, CD151, CD44v6, and integrins CD49c and CD49d, as well as intersectin and clathrin, have been shown to be the main interacting partners of Tspan8 13. In colorectal cancer, Tspan8 regulates cell motility and p120‐catenin function by interaction with E‐cadherin 14; recently, interaction of Tspan8 with mephrin‐β was demonstrated 15. Furthermore, targeting Tspan8 function may offer benefit as a therapeutic intervention in ovarian 16 and colorectal 17 carcinomas, hence attracting attention to this molecule as a potential drug target. Recently, Tspan8 was identified as a biomarker for a subset of deeply quiescent mammary stem cells (Lgr5^+^Tspan8^hi^), whose transcriptome resembled claudin‐low breast carcinoma 18, thus indicating a potential role in breast cancer. In this study, we analysed the expression of Tspan8 in breast cancer in primary lesions and in metastases in humans and examined its impact on the EMT–MET programme in breast cancer cells and on the release and function of extracellular vesicles using a syngeneic rat breast cancer model.

## Materials and methods

### Patient samples

Biopsies were acquired from breast cancer patients and normal breast samples were obtained from individuals undergoing plastic surgery. All samples were taken after the study had been approved by the local ethics committee and written informed patient consent had been obtained. Immunohistochemistry was performed as described in supplementary material, Supplementary materials and methods.

### *In vivo* model

Animal experiments were performed according to regional and national guidelines and approved by the ethics committee. Orthotopic injection of female Fischer rats F344/DuCrl (Charles River, Sulzfeld, Germany) was performed into a mammary fat pad (*n* = 5 per group, 1 × 10^6^ cells). Animals were euthanised after 18 days; organs were prepared to control metastases, and blood was used for extracellular vesicle (EV) preparation as described in supplementary material, Supplementary materials and methods.

### Cell lines and cell assays

Detailed information regarding cultivation of different cell lines under 2D and 3D conditions is provided in supplementary material, Supplementary materials and methods.

### AFM‐based single‐cell force spectroscopy

A detailed description of the method is provided in supplementary material, Supplementary materials and methods.

### Immunofluorescence

Cells were grown in eight‐chamber slides (Ibidi, Planegg, Germany), fixed, permeabilised, blocked and incubated with a primary antibody (60 min, 4°C), washed three times, incubated with a fluorochrome‐conjugated secondary antibody (60 min, 4°C), mounted with Dako mounting medium, and analysed using an LSM510 confocal microscope (Carl Zeiss, Oberkochen, Germany).

### Preparation of EVs

EVs were isolated and characterised according to the MISEV requirements 19 as described previously 13 (supplementary material, Supplementary materials and methods; EV‐TRACK database number MG3779JM).

### Protein and RNA analysis

A detailed description of the method is provided in supplementary material, Supplementary materials and methods.

### Statistics

All experiments were carried out in at least triplicates (regardless of intra‐assay triplicates) and statistically evaluated by the two‐tailed Student's *t*‐test or Mann–Whitney. *P* values of less than 0.05 were considered significant.

## Results

### Tspan8 is expressed in breast cancer primary lesions and in metastases in different organs

**Figure 1:**
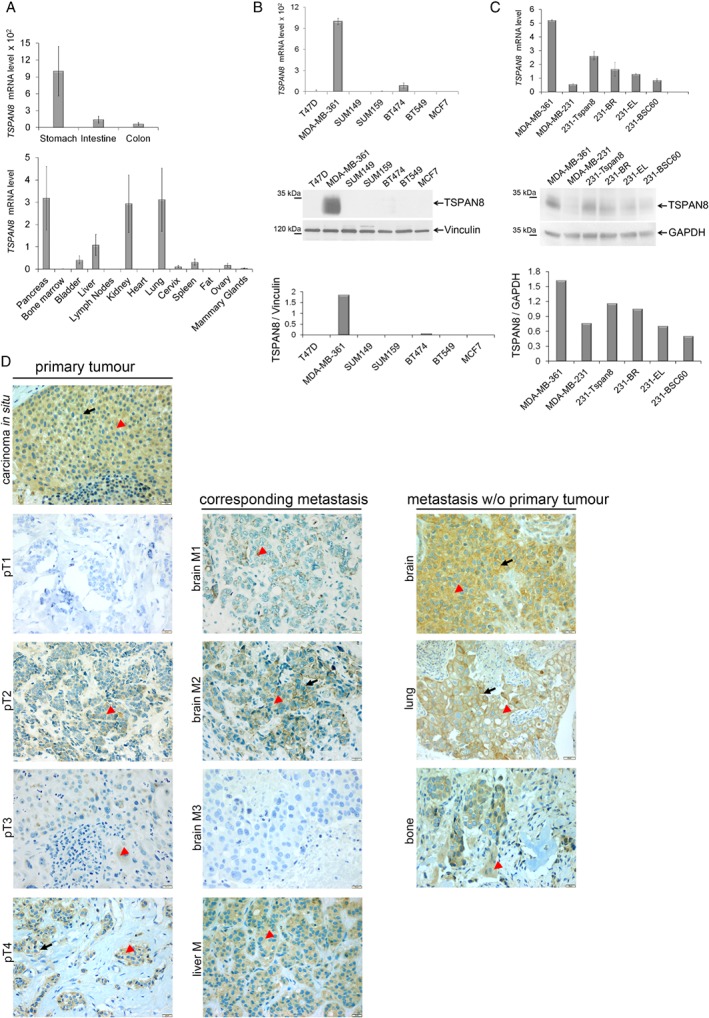
Analysis of TSPAN8 expression in breast cancer cell lines and human tumours. (A) *TSPAN8* mRNA expression was tested in different organs in mice using RT‐PCR. Highest expression was detected in the organs of the digestive system – stomach, intestine, and colon – which exhibited 100‐fold higher expression than in the other organs tested (see values on the *y*‐axis). (B) TSPAN8 expression was examined on RNA (upper panel) and protein levels (bottom panel) in seven breast cancer cell lines; *GAPDH* and vinculin were used as loading controls for RNA and proteins, respectively. TSPAN8 was strongly expressed only in the MDA‐MB‐361 cells derived from brain metastases. (C) MDA‐MB‐231 cell model, consisting of MDA‐MB‐231 parental cell line, 231‐BR brain‐seeking clone, and BSC60 bone‐seeking clone, was tested for *TSPAN8* (upper panel) and TSPAN8 protein (bottom panel) expression. The 231‐Tspan8 cells – parental cell line stably transfected with Tspan8, and MDA‐MB‐361‐expressing endogenous Tspan8 were used as positive controls. Tspan8 revealed strongest expression in the 231‐BR cells. QPCR was performed in technical duplicates and biological triplicates; WB was repeated twice on independent preparations. (D) Immunohistochemistry of human samples for TSPAN8 was performed on paraffin blocks and representative specimens were chosen: on the upper panel, carcinoma *in situ* shows cytoplasmic localization of TSPAN8; four pairs of primary tumours (pT1, pT2, pT3, pT4) and corresponding metastases (brain M1, brain M2, brain M3, liver M4) originating from the same patient exhibit heterogeneous staining of TSPAN8 in primary tumours and in metastases. Right panel: a brain metastasis with strong membrane and cytoplasmic TSPAN8 staining, a lung metastasis with strong membrane and cytoplasmic staining, and a bone metastasis with cytoplasmic TSPAN8 staining are demonstrated. Arrowheads indicate cytoplasmic TSPAN8 and arrows membrane TSPAN8. Scale bar = 20 μm.

While the majority of tetraspanins are ubiquitously expressed, in a healthy organism, the presence of TSPAN8 is mainly restricted to the digestive tract (Figure 1A) 20. However, TSPAN8 is likely to be up‐regulated in different cancer types 21. To address the possibility of TSPAN8 playing a role in breast cancer, we first tested seven breast cancer cell lines (supplementary material, Table S4) 22 for TSPAN8 expression and observed that only MDA‐MB‐361 cells derived from brain metastases exhibited high levels of TSPAN8. Other cell lines originating from primary tumours or pleural effusions were found to be Tspan8^−^/Tspan8^low^ (Figure 1B). We also tested mRNA isolated from cancer patient samples and detected different levels of TSPAN8‐coding mRNA in 4/7 primary tumours and 6/7 lymph node metastases, further supporting a role for TSPAN8 in breast cancer (supplementary material, Figure S1). Subsequently, we examined the MDA‐MB‐231 model, consisting of the parental cell line and two derivatives – 231‐BR cells, representing a brain‐seeking clone 23, and 231‐BCS60 cells, representing a bone‐seeking clone 24. In this model, TSPAN8 was expressed significantly higher in the brain‐seeking 231‐BR cells than in the parental and the 231‐BSC60 cells (Figure 1C), indicating that expression of TSPAN8 may vary in the primary tumour and its metastases.

These first observations prompted us to perform immunohistochemistry in 27 primary human tumours (16 ductal carcinomas, seven lobular carcinomas, and four carcinomas *in situ*) and in 53 metastases from different organs (30 brain, nine liver, eight bone, and six lung metastases). TSPAN8 was detected in 24/27 primary lesions and in 41/53 metastases; specifically, 18/30 brain metastases and all of the liver, bone, and lung metastases showed TSPAN8‐specific staining (Table 1 and Figure 1D). The specimens exhibited a heterogeneous signal and by 5–80% positive cells, the samples were considered positive.

**Table 1:** Immunohistochemistry for Tspan8 in breast cancer primary tumours and in their metastases

Primary tumour No	Primary tumour	TSPAN8 cytoplasmic	TSPAN8 membrane	Metastasis No	Metastasis	TSPAN8 cytoplasmic	TSPAN8 membrane
1	Poorly differentiated, invasive ductal	50% +	–	1	Brain	20% +	10% +
2	Poorly differentiated, invasive ductal	50% +	–	2	Brain	–	–
3	Poorly differentiated, invasive ductal	–	–	3	Brain	10% +	–
4	Poorly differentiated, invasive ductal	70% +	–	4	Brain	–	–
5	Poorly differentiated, invasive ductal	–	–	5	Brain	–	–
6	Moderately differentiated, invasive ductal	–	–	6	Brain	–	–
7	Moderately differentiated, invasive ductal	80% +	–	7	Brain	–	10% +
8	Moderately differentiated, invasive ductal	–	–	8	Brain	–	–
9	Poorly differentiated, lymphangio‐invasive	80% +	–	9	Brain	40% +	10%
10	Moderately differentiated, invasive ductal	80% +	single +	10	Liver	80% +	–
11	Moderately differentiated, NST	80% ++	–	11	Liver	80% +	–
12	Poorly differentiated, invasive ductal	80%+	10%+	12	Liver	80% +	–
13	Moderately differentiated, invasive lobular	90%++	single +	13	Bone	50% + **/***	–
14	Moderately differentiated, invasive lobular	95%+++	–	14	Bone	90% + */***	–
15	Moderately differentiated, invasive ductal	80%++	–		No information		
16	Well differentiated, NST	80%+	–		No information		
17	Well differentiated, invasive ductal	80%++	–		No information		
18	Moderately differentiated, NST	80%++	5%		No information		
19	Moderately differentiated, NST	80%+	–		No information		
20	Poorly differentiated, invasive ductal	80%+	20%+		No information		
21	Moderately differentiated, invasive lobular	80%+	single +		No information		
22	Moderately differentiated, invasive lobular	80%+	–		No information		
23	Moderately differentiated, invasive lobular	80%++	–		No information		
24	DCIS (G2)	80%+	–				
25	DCIS (G2)	80%+	20%+				
26	DCIS (G2)	80%+	20%+				
27	DCIS (G2)	80%+	single +				
	Moderately differentiated, invasive ductal			15	Brain	50% +	–
	Moderately differentiated, invasive ductal			16	Brain	–	–
	Poorly differentiated, invasive ductal			17	Brain	–	–
	Moderately differentiated, invasive ductal			18	Brain	–	–
	Moderately differentiated, invasive lobular			20	Brain	–	–
	Moderately differentiated, invasive lobular			21	Brain	–	–
	Moderately differentiated, invasive lobular			22	Brain	50% +	50% ++
	Moderately differentiated, invasive lobular			23	Brain	–	–
	Moderately differentiated, invasive lobular			25	Brain	–	–
	No information			26	Brain	80% +++	40% ++
	No information			27	Brain	80% +	5% +
	No information			28	Brain	80% +	–
	No information			29	Brain	80% +	–
	No information			30	Brain	80% ++	5% +
	No information			31	Brain	–	–
	No information			32	Brain	80% +	–
	No information			33	Brain	80% +	–
	No information			34	Brain	80% +	–
	No information			35	Brain	80% +	–
	No information			36	Brain	80% ++	–
	No information			37	Brain	80% +	5% +
	Invasive lobular			38	Liver	80% +	–
	Invasive ductal			39	Liver	80% +	–
	No information			40	Liver	80% +	–
	No information			41	Liver	80% +	–
	No information			42	Liver	80% +	5% +
	No information			43	Liver	80% +	Single +
	Invasive NST			44	Lung	80% +	10% +
	Invasive, NST			45	Lung	80% ++	50% +
	No information			46	Lung	80% +	50% +
	No information			47	Lung	80% +	–
	No information			48	Lung	80% +	–
	No information			49	Lung	80% +	–
	Moderately differentiated, invasive NST			49	Bone	80% +	Single +
	Moderately differentiated, invasive ductal			50	Bone	80% +	30% +
	No information			51	Bone	80% +	–
	No information			52	Bone	80% +	–
	No information			53	Bone	80% +	Single +
	No information			54	Bone	80% +	10% +

Data for TSPAN8 expression in the primary tumours and their corresponding metastases detected in the same patient are linked horizontally. Only analysed specimens are numbered: thus, there are 27 primary tumours, and 54 metastases were examined.

70% +: 70% epithelium stained at low intensity; −: no TSPAN8 staining was detected.

Intensity was estimated as +, low; ++, moderate; and +++, strong by three pathologists.

It should be mentioned that to test for a link between TSPAN8 expression in primary tumours and the corresponding metastases, we were able to use 14 samples where both the primary tumour and the metastases originated from the same patient (Table 1). Thus, nine patients with brain metastases, three with liver metastases, and two with bone metastases were examined (Table 1 and Figure 1D). We observed that the expression levels of TSPAN8 in primary tumours and in the corresponding metastases differed from each other, indicating that expression thereof may change during tumour progression.

### Overexpression of Tspan8 enhances cell–cell adhesion and proliferation

**Figure 2:**
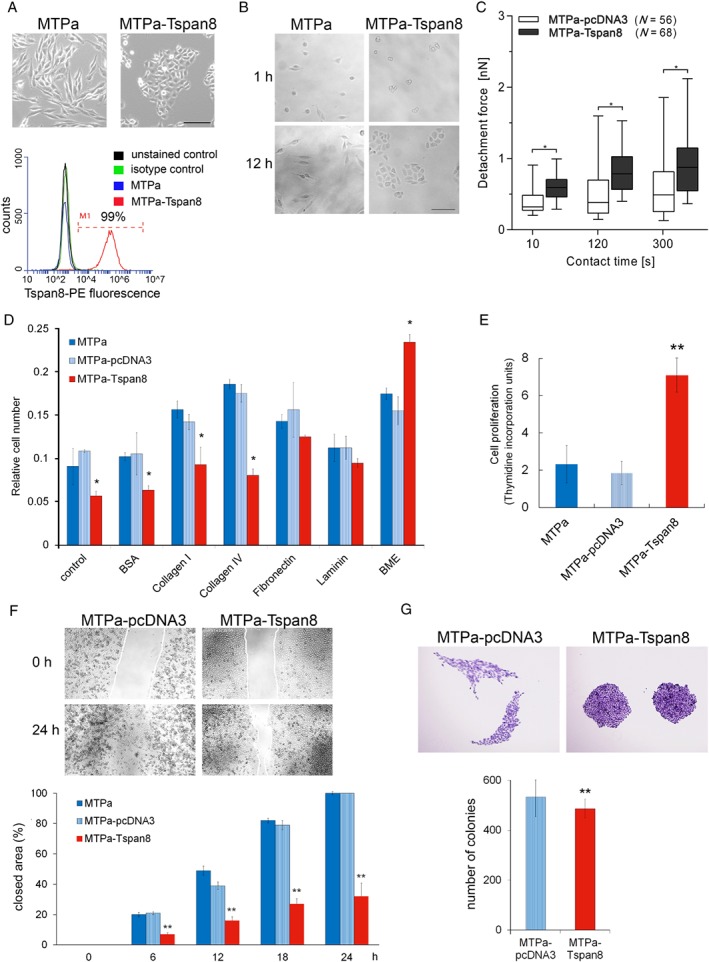
Tspan8 supports proliferation and cell–cell adhesion in breast cancer. (A) Upper panel: stable expression of Tspan8 in a rat breast cancer cell line MTPa leads to changes in cell morphology (scale bar = 200 μm). Bottom panel: Tspan8 surface expression was assessed using flow cytometry, which revealed 99% Tspan8^+^ cells. (B) Impact of Tspan8 on cell clustering was assessed. Cells were seeded at low density and monitored for 12 h. The experiment was repeated three times. (C) Cell–cell adhesion was tested using atomic force microscopy. Tspan8‐expressing cells exhibited significantly higher adhesion forces than cells transfected with a control pcDNA3 plasmid. The analysis on a single cell level revealed a high level of heterogeneity, showing strong variability of adhesive properties of single cells, resulting in high error bars calculated as a standard error by statistical analysis. (D) Cell–matrix adhesion was tested on different substrates using pre‐coated 24‐well plates. Tspan8 expression resulted in a significant reduction of cell–matrix adhesion when no additional coating was used and on the collagen I and IV matrixes; the BME coating resulted in a significant increase of cell–matrix adhesion of the MTPa‐Tspan8 cells. (E) To test cell proliferation, [^3^H]thymidine incorporation was measured. Tspan8 strongly supported cell proliferation. (F) Migration was tested using time‐lapse microscopy. Confluent cultures of the MTPa‐pcDNA3 and MTPa‐Tspan8 cells were used to produce a ‘wound’. Wound closure was observed for 24 h. Videos are available in supplementary material, Movies S1 and S2. Quantitative analysis of cell‐free areas performed using ImageJ Plugin (right panel) showed that Tspan8 strongly reduces cell migration over the ‘wound’ (scale bar = 1000 μm). The experiment was performed in biological duplicates and technical triplicates. *p* < 0.001 was considered as highly significant. (G) Colony‐forming ability was assessed by seeding 100 cells per 10 cm dish. While no significant difference in colony size was observed, Tspan8 mediated a slight but significant reduction of colony‐forming ability (scale bar = 500 μm).

**Figure 3:**
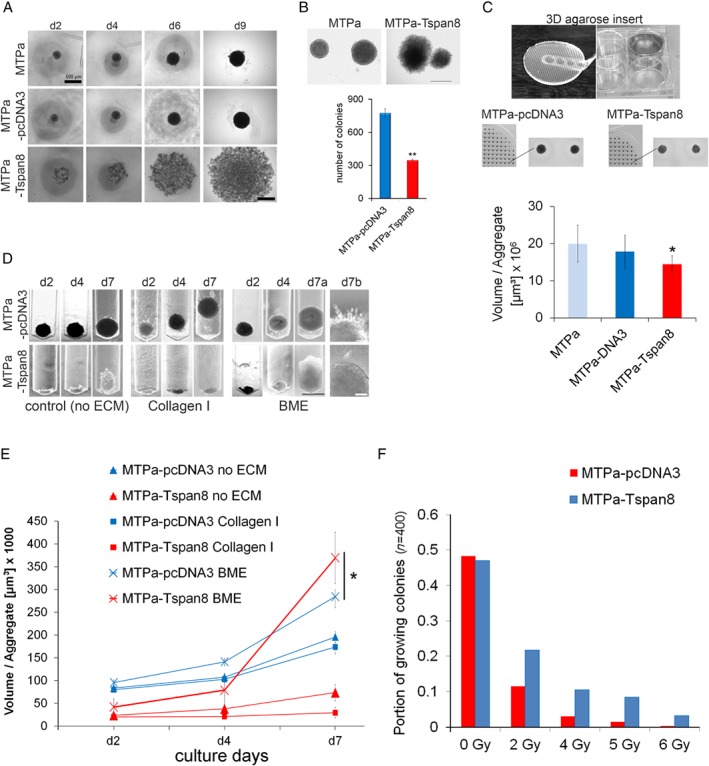
Tspan8 supports proliferation in BME ECM and mediates radiation resistance in a 3D environment. (A) To characterise aggregate formation, 1000 cells per well were seeded in a 48‐well plate coated with 1.5% agarose gel and monitored for 9 days. MTPa cells formed tight cell aggregates, whereas the MTPa‐Tspan8 cells lost their ability for cell–cell contact and formed loose cell aggregates (scale bar = 200 μm). (B) Anchorage‐independent growth was tested using soft agar colony formation assay. MTPa‐Tspan8 cells formed about 300 colonies, whereas nearly 800 colonies were counted for MTPa‐pcDNA3 cells, showing a strong negative effect of Tspan8 on anchorage independence (scale bar = 500 μm). (C) A microwell array in six‐well plates (upper panel) was used to test cell proliferation under 3D conditions. For quantitative analysis, MTT staining (reflecting metabolic activity of the cells) was used (bottom panel). It showed a slight reduction of proliferation/metabolic activity of MTP‐Tspan8 cells compared with the MTPa‐pcDNA3 cells (scale bar = 1000 μm). (D) Cell invasion was tested in customised inserts (supplementary material, Figures S3 and S4) for 7 days without ECM (left panel), in collagen I (middle panel), and in BME (right panel, scale bar = 500 μm). (E) Quantitative analysis of cell invasion revealed that in BME, MTPa‐Tspan8 cells exhibit a significantly stronger proliferation than the parental MTPa‐pcDNA3 cells, showing an increased growth rate after day 4. (F) Gamma‐irradiation resistance of 3D cell aggregate was tested using ^137^Cs with 0.66 Gy/min on day 2 as described in supplementary material, Figure S5. A significantly higher number of the MTPa‐Tspan8 aggregates grew upon irradiation than the MTPa‐pcDNA3 aggregates. All experiments, if not otherwise mentioned, were repeated at least three independent times.

To assess the direct impact of Tspan8 on breast cancer growth and progression, we chose a syngeneic rat breast cancer model. Overexpression of Tspan8 in the MTPa 25 parental Tspan8‐negative cells led to the formation of compact cell clusters (Figure 2A). We seeded the MTPa‐pcDNA3 and MTPa‐Tspan8 cells by low density and tracked cluster formation for 12 h. Only MTPa‐Tspan8 cells established clusters, suggesting that Tspan8 supports intercellular adhesiveness (Figure 2B). Measurement of cellular adhesion forces using atomic force microscopy by cell retraction 26, 27 supported this observation and showed that the MTPa‐Tspan8 cells exhibit significantly stronger cell–cell adhesion compared with the MTPa‐pcDNA3 cells (Figure 2C). In contrast, MTPa and MTPa‐pcDNA3 cells displayed faster cell–matrix adhesion to collagens I and IV, while the MTPa‐Tspan8 cells adhered significantly faster to BME (human basement membrane extract) (Figure 2D). The MTPa‐Tspan8 cells exhibited approximately three‐fold higher efficiency of thymidine incorporation, indicating that Tspan8 also supports cell division (Figure 3E). A similar effect of Tspan8 was observed in the MDA‐MB‐231 cells stably transfected with Tspan8, indicating the similarity of Tspan8 action in rats and humans (supplementary material, Figure S2).

In cell monolayers wounded by scraping the surface, wound closure was significantly delayed in the MTPa‐Tspan8 cells compared with the parental and MTPa‐pcDNA3 cells (Figure 2F). Documentation of wound healing by video microscopy revealed that whereas single cells of MTPa and MTPa‐pcDNA3 may abandon the cell population and migrate over the scratch, some of the MTPa‐Tspan8 cells migrate along the population boundary, while the majority of cells maintain cell–cell contacts, suggesting that Tspan8 impairs wound closure by supporting cell–cell adhesion and thereby preventing scattering of the cell colonies (supplementary material, Movies S1 and S2).

Additionally, we compared the colony formation ability of MTPa‐pcDNA3 versus MTPa‐Tspan8 cells to determine the impact of Tspan8 on the self‐sufficiency of a single cell. Performed under 2D conditions, MTPa cells formed colonies of irregular shape and loose cell–cell contact; in contrast, the MTPa‐Tspan8 cells formed tight round colonies, adding strength to the hypothesis that Tspan8 plays a role in cell–cell adhesion (Figure 2G, upper panel). Statistical analysis demonstrated a slightly higher colony‐forming ability of the MTPa‐pcDNA3 cells compared with the MTPa‐Tspan8 cells (Figure 2G, bottom panel), suggesting that Tspan8 does not enhance the self‐sufficiency of tumour cells under conventional cell culture conditions.

### Under 3D conditions, Tspan8 supports cell–cell adhesion and proliferation in the presence of basal membrane extract (BME), and mediates radiation resistance

To test Tspan8 function under conditions more closely resembling the physiological environment, we employed conventional and customised 3D cell cultures. To examine the ability of the cells for anchorage‐independent growth, an agarose liquid overlay was used. The MTPa and MTPa‐pcDNA3 cells formed compact spheroids, whereas the MTPa‐Tspan8 cells – in contrast to their behaviour under 2D conditions – formed large aggregates with loose cell–cell contacts, indicating impairment of the Tspan8 ability to support cell–cell contact under the conditions being tested (Figure 3A). Similar morphology of the colonies was observed in a conventional soft agar assay (Figure 3B, upper panel); quantitative analysis revealed that Tspan8 overexpression causes a strong reduction of the colony number (Figure 3B, bottom panel), indicating diminished resistance to anoikis by the MTPa‐Tspan8 cells. Using recently characterised agarose microwell arrays 28, we tested cell proliferation and observed that, in contrast to 2D, the Tspan8 proliferation‐supporting effect was attenuated in a 3D environment (Figure 3C).

Cancer progression involves ECM remodelling and, vice versa, the ECM may mediate cancer cell behaviour 29. To address the impact of different ECMs on Tspan^+^ and Tspan8^−^ cells, we employed a customised agarose device allowing analysis of cell growth and behaviour in a 3D environment inaccessible by conventional transmigration assays, allowing quantification of cell invasion in 2D conditions (supplementary material, Figure S3). Collagen I, which is the most abundant bone ECM constituent 30, and BME, a cell culture‐derived basal membrane extract consisting of laminin, collagen IV, and proteoglycans, and resembling the basal lamina of internal organs, including the brain 31, were chosen for the analysis. The MTPa cells formed compact, rapidly proliferating aggregates in the control matrixes, whereas the MTPa‐Tspan8 cells formed slow‐growing clusters with loose cell–cell contacts (Figure 3D, left panel). In the presence of collagen I, the MTPa, but not the MTPa‐Tspan8, cell spheroids formed outgrowths and moved along the cavity, indicating a potential for invasiveness (Figure 3D, middle panel and supplementary material, Figure S3). The MTPa‐Tspan8 cells formed compact aggregates with a diminished proliferation ability (Figure 3D, middle panel). However, when the cells were seeded into the BME, the MTPa‐Tspan8 cells exhibited a significantly stronger proliferation than MTPa (Figure 3D, right panel; 3E), further supporting the importance of specific ECM components for Tspan8 function observed under 2D conditions.

Radiation resistance is the main obstacle to therapeutic success in breast cancer metastases 32. To address the potential association of Tspan8 with radiation resistance, we tested the colony‐forming ability of the MTPa‐pcDNA3 and MTPa‐Tspan8 cells upon exposure to increasing radiation doses in 3D microwell array. Tspan8 strongly enhanced the resistance of cells to irradiation, mediating the survival of a higher number of colonies (Figure 3F and supplementary material, Figure S4). Similar results were obtained in human breast cancer cell lines when a radiosensitivity clonogenic survival assay in 2D culture described elsewhere 33, 34 was performed (supplementary material, Figure S5). These data supported a role of Tspan8 in radiation resistance in breast cancer.

### Tspan8 mediates a reversible change of cell phenotype resembling the mesenchymal–epithelial transition (MET)

**Figure 4:**
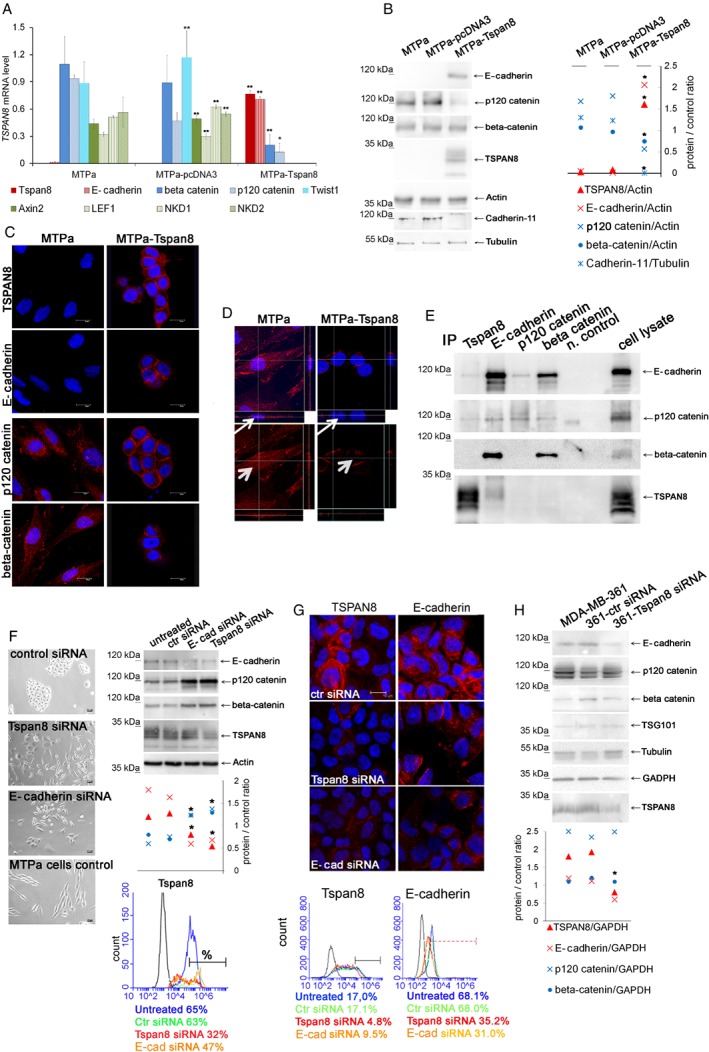
Tspan8 induces mesenchymal–epithelial transition. (A) Total RNA was harvested from MTPa, MTPa‐pcDNA3, and MTPa‐Tspan8 cells, and expression of mRNA coding for E‐cadherin, β‐catenin, p120‐catenin, Twist, and the β‐catenin target genes *Axin2*, *LEF1*, *NKD1*, and *NKD2* was analysed by RT‐qPCR. *GAPDH* was used as a reference transcript. Overexpression of Tspan8 resulted in up‐regulation of E‐cadherin*,* highly significant down‐regulation of β‐catenin and p120‐catenin, and complete abrogation of Twist1, Axin2, LEF1, NKD1, and NKD2 expression. Technical duplicates and biological triplicates were analysed. (B) Cell lysates were prepared from MTPa, MTPa‐pcDNA3, and MTPa‐Tspan8 cells. WB analysis confirmed the presence of E‐cadherin. Densitometry analysis of protein signal intensity performed by ImageJ revealed significantly diminished levels of p120‐catenin and cadherin‐11 in the MTPa‐Tspan8 cells. No significant regulation of β‐catenin at the protein level was observed. The experiment was repeated five times. (C) Immunofluorescence analysis of the MTPa and MTPa‐Tspan8 cells. Cells were cultured for 24–48 h in chamber slides, fixed, and stained with the indicated antibodies. (D) Z‐stack of β‐catenin staining was generated for MTPa and MTPa‐Tspan8 cells. Red β‐catenin‐specific fluorescence was detected in the cytoplasm and in the nuclei of MTPa cells, but not of MTPa‐Tspan8 cells (small arrow in the Z‐stack and arrowhead in the *x/y* flat bottom image). Immunofluorescence was assessed four times independently. (E) Co‐immunoprecipitation was performed in the MTPa‐Tspan8 cell lysates using antibodies specific to Tspan8, E‐cadherin, p120‐catenin, and β‐catenin. As a negative control, cell lysates were incubated with the corresponding isotype control mouse IgG and protein G Sepharose. The experiment was performed three times. (F) Rescue of Tspan8‐induced MET was tested by Tspan8 and E‐cadherin knockdowns. Bright‐field images were taken 72 h after siRNA transfection of MTPa‐Tspan8 cells with siRNA. To assess knockdown, proteins were harvested 48 h post‐transfection, analysed by WB (right upper panel) and quantified using ImageJ (right middle panel). To assess surface expression, flow cytometry was performed 48 h after transfection (bottom panel). (G) Knockdown of Tspan8 and E‐cadherin was performed in MDA‐MB‐361 cells followed by immunofluorescence and flow cytometry. Heterogeneous Tspan8 staining was observed in cells transfected with control siRNA, and siRNAs specific for Tspan8‐ and E‐cadherin. The experiment was performed three times and representative images are shown. Changes in Tspan8 and E‐cadherin surface expression after siRNA were assessed by flow cytometry (bottom panel). The proportion of Tspan8 strongly‐positive cells was calculated. The experiment was repeated twice; representative diagrams are shown. (H) MDA‐MB‐361 cells were transfected with scramble‐siRNA and Tspan8‐siRNA; proteins were harvested 48 h post‐transfection and analysed by WB followed by densitometry analysis using ImageJ. Both Tspan8 and E‐cadherin were significantly down‐regulated, while neither β‐catenin nor p120‐catenin was affected (bottom panel).

To understand the molecular mechanisms behind the observed phenomena, we analysed the signalling pathways involved in the mechanisms of the Tspan8 action. Because the phenotypical changes mediated by Tspan8 resembled mesenchymal–epithelial transition (MET), we tested for the expression of genes coding for Twist, E‐cadherin, β‐catenin, and p120‐catenin as the main regulators of the EMT–MET programmes 1. As shown in Figure 4A, Tspan8 overexpression mediated significant up‐regulation of E‐cadherin, which is characteristic for epithelial cells, while Twist, β‐catenin, and p120‐catenin, which are associated with mesenchymal cell morphology, were significantly down‐regulated. Furthermore, expression of the β‐catenin target genes *Axin2*, *LEF1*, *NKD1*, and *NKD2* 35 was completely abolished in the MTPa‐Tspan8 cells, indicating modulation of β‐catenin signalling upon Tspan8 overexpression (Figure 4A). On the protein levels, E‐cadherin and Tspan8 were significantly increased, while p120‐catenin and cadherin‐11, the regulators of mesenchymal cell properties 36, were significantly decreased in the MTPa‐Tspan8 cells, supporting the notion that Tspan8 mediated MET (Figure 4B). Using immunofluorescence, we observed membrane localisation of Tspan8 and E‐cadherin in the MTPa‐Tspan8 cells (Figure 4C). While p120‐catenin and β‐catenin exhibited cytoplasmic localisation in the MTPa cells, they were detected on the cell membrane in the MTPa‐Tspan8 cells (Figure 4C). Additionally, β‐catenin‐specific signals were observed in the nuclei of the MTPa, but not MTPa‐Tspan8, cells (Figure 4D, white arrows), which together with the observed down‐regulation of the β‐catenin target genes in MTPa‐Tspan8 cells further supported Tspan8‐mediated modulation of the β‐catenin signalling pathway. Pronounced membrane staining of Tspan8, E‐cadherin, and catenins prompted us to test their potential interaction. Co‐immunoprecipitation revealed E‐cadherin‐ and p120‐catenin‐specific bands in the Tspan8 precipitate. Tspan8 was also detected in the E‐cadherin precipitate together with p120‐catenin and β‐catenin, indicating their association (Figure 4E).

Rescue of Tspan8‐induced MET was tested by Tspan8 and E‐cadherin knockdown. Partial reversion of the epithelial cell morphology was observed 72 h after transfection with Tspan8‐ and E‐cadherin‐specific RNA (Figure 4F, left panel). To control knockdown, western blotting (WB) (Figure 4F, right upper panel) and flow cytometry were performed (Figure 4F, right bottom panel). Significant down‐regulation of Tspan8 and E‐cadherin upon transfection with Tspan8‐ and E‐cadherin siRNAs was observed, indicating their co‐regulation; this was further supported by up‐regulation of p120‐catenin and β‐catenin in both cases. Flow cytometry was performed 48 h after transfection to examine surface expression of Tspan8 (Figure 4F, right bottom panel). The untreated cells and cells transfected with control scrambled siRNA showed 65% and 63% Tspan8 surface expression, respectively (Figure 4F, right bottom panel, blue line, green line). Following Tspan8 siRNA (red line) and E‐cadherin siRNA (orange line), the number of positive cells was reduced to 32% and 47%, respectively. These data were consistent with the gene expression analysis (supplementary material, Figure S6).

Next, we tested co‐regulation of TSPAN8 and E‐cadherin in MDA‐MB‐361 human breast cancer cells expressing endogenous TSPAN8 and E‐cadherin. Knockdown of TSPAN8 and E‐cadherin was performed in MDA‐MB‐361 cells and examined by immunofluorescence and flow cytometry (Figure 4G). Heterogeneous TSPAN8 staining was observed in cells transfected with control siRNA, and siRNAs specific for Tspan8 and E‐cadherin. Strong heterogeneity of fluorescence signals hampered their quantification. Therefore, changes in TSPAN8 and E‐cadherin surface expression following siRNA treatment were assessed by flow cytometry (Figure 4G, bottom panel). The portion of TSPAN8‐strongly positive cells (Tspan8^++^/MFI > 10^5^) was reduced after siRNA transfection: 17% Tspan8^++^ cells were detected in non‐transfected cells (blue line), 4.8% in Tspan8‐siRNA transfected cells (red line), and 9.5% in E‐cadherin (orange line) transfected cells. The proportion of E‐cadherin‐positive cells (MFI > 10^3^) comprised 68.1% and 68% in the non‐transfected cells (blue line) and cells transfected with the control siRNA (green line), respectively. E‐cadherin surface expression was reduced to 35.2% and 31% after transfection with Tspan8 (red line) and E‐cadherin‐siRNA (orange line), respectively. Co‐regulation of Tspan8 and E‐cadherin was also observed at the RNA level, showing that E‐cadherin is significantly reduced after Tspan8 knockdown and, vice versa, Tspan8 was reduced after E‐cadherin knockdown (supplementary material, Figure S7). However, in contrast to the MTPa cells, neither p120‐catenin nor β‐catenin was affected by the Tspan8 or E‐cadherin siRNAs in the MDA‐MB‐361 cells (Figure 4H).

### Tspan8 supports formation of metastases and EV release *in vivo*

**Figure 5:**
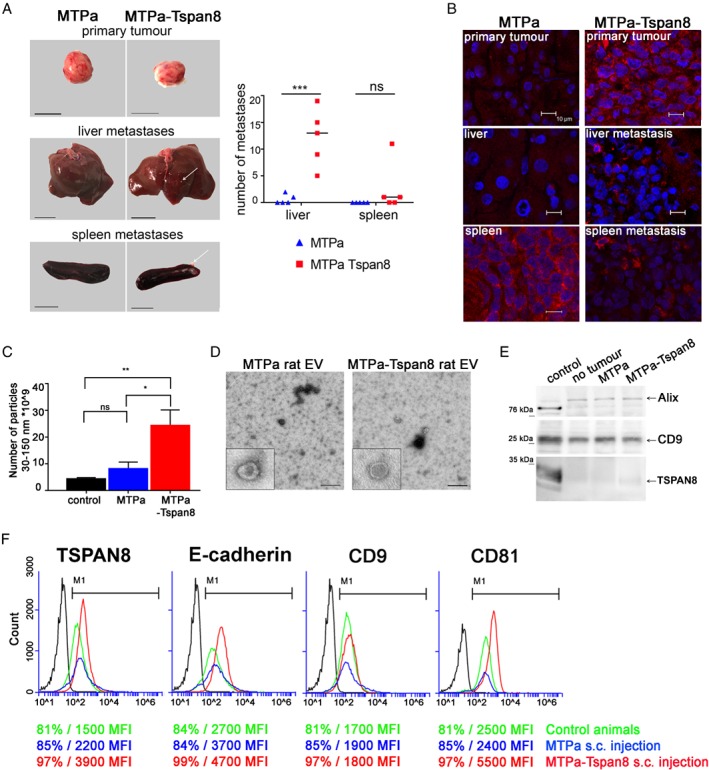
Tspan8 supports metastases and mediates a significant increase in EV number in the circulation *in vivo*. (A) Orthotopic injection of rats was performed using 1 × 10^6^ MTPa or MTPa‐Tspan8 cells per animal; cells were injected into the mammary fat pad (five animals per group). At day 18, animals were sacrificed and organs isolated. Representative images of primary tumours, liver, and spleen are shown. Pictures were taken and metastases counted using ImageJ. (B) Immunofluorescence was performed on frozen sections of primary tumours and liver and spleen specimens from both the animals harbouring MTPa‐ and those harbouring MTPa‐Tspan8 tumours. (C) EVs were collected from rat blood and measured by NTA. Significantly higher numbers of EV were detected in the blood of rats injected with MTPa‐Tspan8 cells. (D) Transmission electron microscopy of EV preparations revealed vesicular structures typical for EVs isolated from blood. (E) EVs were lysed and equal amounts of proteins were loaded for WB analysis and tested with CD9, Alix, and Tspan8 antibodies. No differences in CD9 and Alix protein amounts were observed in control animals and in animals harbouring MTPa or MTPa‐Tspan8 tumours. (F) Flow cytometry analysis of EVs. Tspan8, CD9, and CD81 were tested and the percentage of positive EVs and the MFI value were counted. Strong differences in the MFI values between the origin of EVs from control animals (green line) and the origin of EVs from MTPa (blue line) and MTPs‐Tspan8 (red line) animals were noticed, showing a strong increase in the MFI of Tspan8 and CD81 in EVs derived from MTPa‐Tspan8 animals. Flow cytometry was performed twice and representative images are shown.

Next, we tested the effect of Tspan8 on the tumourigenic properties of breast cancer cells *in vivo*. The MTPa and MTPa‐Tspan8 cells were injected orthotopically into mammary fat pads of Fischer rats. No differences in the growth of primary tumours were observed (Figure 5A, upper panel). However, the MTPa‐Tspan8 tumours formed multiple metastases in the liver and spleen, suggesting a role for Tspan8 in metastases in breast cancer (Figure 5A). A similar phenomenon was observed when tumour cells were injected intraperitoneally ( supplementary material, Figure S8). Immunohistochemistry showed Tspan8 staining in the MTPa‐Tspan8 but not in the MTPa primary tumours (Figure 5B, upper panel). No Tspan8 signal was observed in the livers of rats harbouring the MTPa tumour (Figure 5B, middle panel); the metastases‐free spleen exhibited strong fluorescence of endogenous Tspan8 (Figure 5B, bottom panel). Weak and heterogeneous Tspan8 fluorescence was observed in the liver and spleen metastases of rats harbouring the MTPa‐Tspan8 tumours (Figure 5B, right panels).

Because extracellular vesicles (EVs) play an important functional role in metastatic cancer progression 37, 38, 39, and Tspan8 has already been shown to function as a modulator of EV release and function 13, we examined the number of EVs in the blood of tumour‐bearing animals. Measurements by nanoparticle tracking analysis (NTA) revealed a several‐fold increase in EV number in the blood of MTPa‐Tspan8 animals (Figure 5C and supplementary material, Figure S9). An even‐stronger effect of Tspan8 was observed after intraperitoneal injection of tumour cells (supplementary material, Figure S10). Transmission electron microscopy revealed vesicles of approximately 100 nm diameter in the EV preparations from the blood of animals harbouring MTPa and MTPa‐Tspan8 tumours (Figure 5D). Testing EVs by WB showed the exosomal markers Alix and CD9 on EVs in each of the samples tested, indicating the presence of exosomes; however, only a very weak Tspan8 signal was detected (Figure 5E). Since tetraspanins undergo strong post‐translational modifications 40, which may hamper protein detection by WB, we applied bead‐assisted EV measurement using flow cytometry 41 to examine Tspan8. We detected Tspan8, CD9, and CD81 signals on EVs isolated in control and in tumour‐bearing animals. The EV level of CD9 was constant, consistent with the WB data; however, the mean fluorescence intensity (MFI) values for Tspan8 and CD81 were strongly increased in EVs isolated from the blood of MTPa‐Tspan8 animals, compared with the MTPa and control animals (Figure 5F and supplementary material, Figure S11), which was consistent with the overall increase in EV number in MTPa‐Tspan8 animals.

### Tspan8 modulates EV release and function *in vitro*

**Figure 6:**
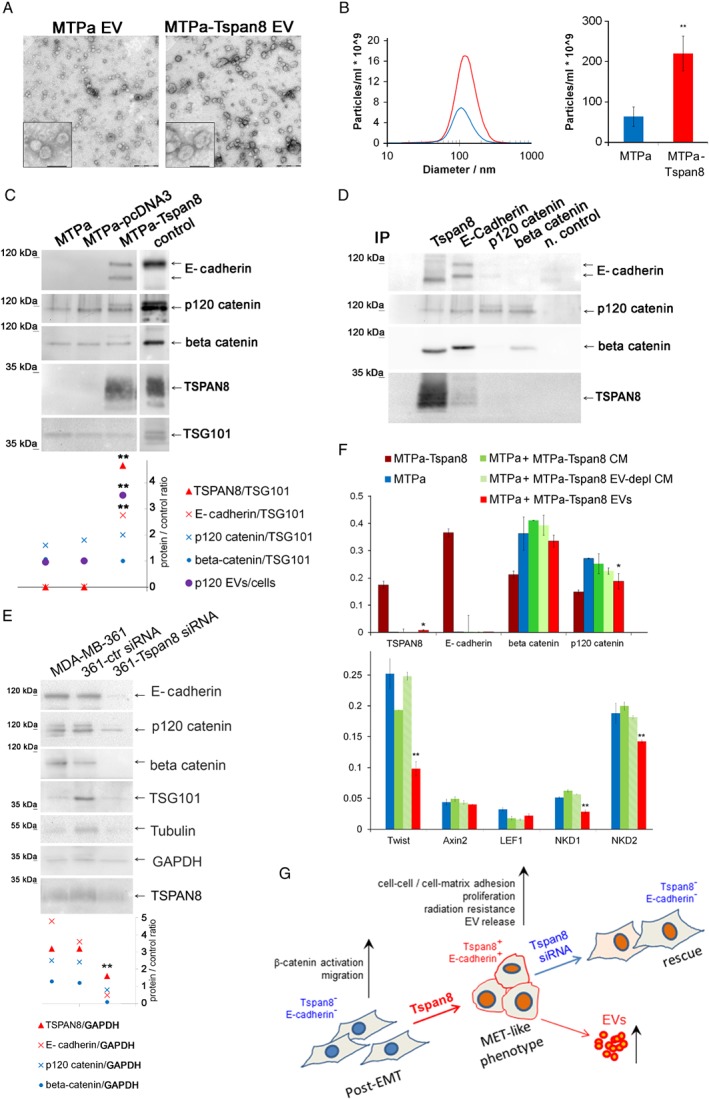
Tspan8 mediates increased EV release *in vitro* and supports recruitment of E‐cadherin and catenins to EVs. (A) Transmission electron microscopy of EVs released by the MTPa and MTPa‐Tspan8 cells. Scale bar equals 500 nm in the large panel and 100 nm in the image with higher magnification. (B) NTA was done with each of the samples isolated and a representative image is shown. Statistical analysis of four independent measurements showed a highly significant increase in EV number (right panel). (C) EVs of MTPa, MTPa‐pcDNA3, and MTPa‐Tspan8 cells were lysed and WB was performed. Densitometry showed a significant increase in the amount of Tspan8, E‐cadherin, and p120‐catenin in EVs of MTPa‐Tspan8 cells; for p120‐catenin, a ratio of protein amount in EVs/protein amount in cells was calculated. (D) Co‐immunoprecipitation was performed in the MTPa‐Tspan8 EVs using antibodies specific to Tspan8, E‐cadherin, p120‐catenin, and β‐catenin. As a negative control, EV lysate was incubated with the corresponding isotype control mouse IgG and protein G Sepharose. The experiment was performed three times. (E) MDA‐MB‐361 cells were transfected either with control siRNA or with Tspan8‐siRNA; EVs were isolated 48 h post‐transfection and analysed by WB followed by densitometry analysis using ImageJ. GAPDH was used as a loading control. (F) Functional analysis with EVs released from the MTPa‐Tspan8 cells was performed. The MTPa cells were treated daily with MTPa‐Tspan8 EVs (5 μg/ml) for 5 days. As controls, MTPa cells were also treated with conditioned cell culture medium (MTPa‐Tspan8 CM) and with EV‐depleted cell culture medium (MTPa‐Tspan8 EV‐depl CM) in order to discriminate between EV‐specific and EV‐non‐specific effects. At day 6, the cells were lysed and RNA was isolated and analysed using RT‐qPCR. Expression of Tspan8, E‐cadherin, β‐catenin, p120‐catenin, Twist, and the β‐catenin target genes *Axin2*, *LEF1*, *NKD1*, and *NKD2* was examined. Significant down‐regulation of p120‐catenin, Twist, NKD1, and NKD2, and up‐regulation of Tspan8 were observed, indicating that MTPa‐Tspan8 EVs may affect features of the MTPa parental cells.

To test directly a role for Tspan8 in the regulation of EV release, we isolated EVs from cultures of MTPa or MTPa‐Tspan8 cells. Electron microscopy showed typical exosome‐like structures of 60–100 nm diameter in EV preparations (Figure 6A). Consistent with the observations *in vivo*, NTA measurements showed a significant increase in the number of EVs from MTPa‐Tspan8 cells compared with MTPa cells. We observed both Tspan8‐ and E‐cadherin‐specific bands in the MTPa‐Tspan8 EVs tested by WB (Figure 6C). Furthermore, recruitment of p120‐catenin to EVs was significantly increased in the presence of Tspan8 (Figure 6C). Therefore, we tested their potential interaction in EVs using co‐immunoprecipitation. Similar to the results in cells, E‐cadherin and p120‐catenin were obtained in the Tspan8 precipitates (Figure 6D), supporting their interaction in EVs. Additionally, interaction between E‐cadherin, p120‐catenin, and β‐catenin was detected. To test a direct contribution by Tspan8 to recruitment of the E‐cadherin/catenin complex to the EVs, we transfected MDA‐MB‐361 cells with control and Tspan8‐specific siRNA (Figure 6E). We observed a significant reduction of E‐cadherin, p120‐catenin, and β‐catenin in EVs after Tspan8 knockdown, further corroborating our hypothesis. The function of these EVs in tumour progression remains to be explored. However, it is noteworthy that treatment of the parental MTPa cells with EVs released by the MTPa‐Tspan8 cells revealed their ability to suppress the expression of p120‐catenin and Twist encoding genes, and to diminish the expression of β‐catenin target genes in the parental cells, adding strength to the hypothesis that Tspan8 impacts the EV amount and may have a role in the regulation of EV content and function in breast cancer (Figure 6F).

## Discussion

On examining TSPAN8 expression in human breast cancer specimens, we observed great variations in the TSPAN8 expression levels within one specimen and between primary lesions and metastases originating from the same patient. Also, in the rat model, the liver metastases revealed heterogeneous Tspan8 staining. This observation is consistent with the overall models of tumour heterogeneity and plasticity explaining variations occurring within one lesion as a consequence of genetic and epigenetic changes during tumour progression, which allows cancer cells to adapt to a changing microenvironment 42. This model is completed by the postulated intra‐tumour hierarchy and cancer stem cells (CSCs) 43, 44. It remains to be explored whether Tspan8 may change the tumourigenic properties of breast cancer CSCs; however, preliminary tests demonstrated that TSPAN8 is not expressed in cultures of the breast cancer stem‐like cells (data not shown) 45. Further investigation will be required to understand the molecular mechanisms of TSPAN8 up‐regulation in breast cancer cells. Hence, several repressors and activators of TSPAN8, such as GSK3β, IQGAP1, TPT1, LCMR1, PTEN, and p53, were recently identified in melanoma 46, 47 which could potentially be involved in the regulation of TSPAN8 expression in breast cancer. Thus, while wild‐type p53 suppresses TSPAN8 expression 46, its somatic mutations occurring in breast cancer 48 may be one of the reasons for the re‐expression of TSPAN8 in a portion of breast cancer cells in primary lesions or metastases. Experimental data will be required to test this hypothesis.

Furthermore, different intracellular locations of TSPAN8 in the cytoplasm and on the membrane were observed, as has already been described for colorectal cancer 14. Another tetraspanin, namely CD151, was reported to exhibit membrane and cytoplasmic localisation in breast cancer 49. Because the intracellular traffic routes and intracellular localisation of tetraspanins are strongly dependent on external stimulation 50, rearrangement of tetraspanin localisation in tumour cells may have functional and, consequently, therapeutic effects. Providing an answer to these questions remains the subject of future investigations.

Here, we addressed the molecular mechanisms of TSPAN8 action in breast cancer cells. Overexpressed in breast cancer cells, Tspan8 induced E‐cadherin expression and mediated a strong down‐regulation of Twist and β‐catenin target genes. Similar to colorectal cancer, co‐immunoprecipitation experiments showed the interaction between Tspan8, E‐cadherin, and p120‐catenin 14. However, whereas Tspan8 is functionally linked to cell migration in colorectal cancer and abrogates E‐cadherin function by collaborating with p120‐catenin and α2β1 integrin 14, in breast cancer Tspan8 is likely to act together with E‐cadherin, regulating cell–cell adhesion and proliferation. Interestingly, the role of E‐cadherin as a tumour suppressor was recently reconsidered because of its ability to support metastatic potential and cell proliferation via the activation of NF‐κB 51. The link between Tspan8 and NF‐κB has not yet been explored. However, also in other cancer types, Tspan8 can act as a regulator of proliferation, motility, and invasiveness 9. Similarly, another tetraspanin, CD151, may exhibit multiple effects including regulation of proliferation, motility, and morphogenesis 52. Furthermore, CD151 – mostly characterised as a tumour‐ and metastasis‐promoting tetraspanin 53 – was also reported to build a complex with the α3β1 integrin, suppressing ovarian tumour growth by inhibiting Slug‐dependent EMT and supporting cell–cell adhesion 54. These data are consistent with the hypothesis that the biological effects of tetraspanins are determined by their interacting partners 55, 56. It seems that Tspan8 mediates cell motility via collaboration with integrins α6β4 in pancreatic cancer 57, α3β1 in gliomas 58, and α2β1 in colorectal cancer 14. Interaction with E‐cadherin resulted in a functional switch of Tspan8 in breast cancer. Alongside increased cell–cell adhesion, Tspan8 supported cell–matrix adhesion to the BME. Since cell–cell and cell–matrix adhesion are functionally linked to each other and involved, alongside cadherins, also integrins 59, a connection between Tspan8 and integrins in breast cancer, as is already known from other cancer types 14, 57, 58, is possible. It may also explain the observed increase of proliferation in the 3D matrix in BME as a physiological response of the cells on signalling across the adhesive network 59.

Addressing the question of the selective advantages mediated by TSPAN8 in breast cancer, we observed that Tspan8^+^ cells exhibit significantly greater radiation resistance compared with control Tspan8^−^ mesenchymal‐like cells, suggesting that MET may also be linked to radiation resistance. In line with this suggestion, up‐regulation of Snail, an EMT trigger, was linked to decreased radiation resistance in MCF7 cells 60. Integrin α6, which is involved in radiation resistance in breast cancer 61, may also be involved in Tspan8‐dependent radiation resistance as its interacting partner 57. Taking into account that cranial radiation remains one of the leading therapies applied for treatment of brain metastases 32, the presence of Tspan8 may be critical for a response to radiation therapy. Consequently, TSPAN8 inhibition, which has been successfully tested for ovarian and colorectal cancer with TSPAN8 inhibitory antibodies 16, 17, may be advantageous for therapy of TSPAN8 metastases.

In addition to regulation of the cell behaviour, Tspan8 mediated a substantial increase in the number of EVs and contributed to the recruitment of its interacting partners to the EVs, consistent with our earlier findings 13. In line with the evidence that Tspan8 may regulate EV content, we have also provided a useful first indication of the function of these EVs, which will, however, require further investigation. Positive results for testing Tspan8 in EVs by flow cytometry and the observed increase in Tspan8 fluorescence intensity in EVs from the blood of Tspan8 tumour‐bearing animals indicate the first hint towards the potential of Tspan8 as a non‐invasive biomarker for breast cancer, which would be useful to address in a clinical study.

To sum up, we have described here the first study on TSPAN8 in breast cancer and have demonstrated the impact of Tspan8 on adhesion, proliferation, radiation resistance, and EV release. First hints towards the molecular mechanisms of Tspan8 action and involvement of E‐cadherin were provided. These results warrant further investigation of Tspan8 in breast cancer towards the understanding of molecular mechanisms and testing the value of Tspan8 as a therapeutic target and a non‐invasive biomarker.

## Author contributions statement

MVo, ART, JN, EG‐N, TG‐S, AG, PJ, FR, CF, CB, MVe, AH, JD, CG, JK, and WT performed experiments and contributed to data analysis and writing the manuscript. BH, GP, GH, CW, CB, AB, TE, CS, and JS contributed to data analysis and writing the manuscript. IN designed and supervised this study, analysed data, and wrote the manuscript.

**SUPPLEMENTARY MATERIAL ONLINE**

**Supplementary materials and methods**

**Figure S1**. RT‐qPCR analysis of TSPAN8 encoding mRNA in human tumours

**Figure S2**. Impact of Tspan8 on adhesion and proliferation of human breast cancer cells MDA‐MB‐231

**Figure S3**. Customised agarose device to test impact of different ECM on cell growth and behaviour

**Figure S4**. Radiation resistance test of MTPa‐pcDNA3 and MTPa‐Tspan8 cells under 3D conditions

**Figure S5**. Impact of Tspan8 on radiation sensitivity using conventional 2D colony formation assays

**Figure S6**. Co‐regulation of Tspan8 and E‐cadherin expression in MTPa‐Tspan8 cells

**Figure S7**. Co‐regulation of Tspan8 and E‐cadherin expression in MDA‐MB‐361 cells

**Figure S8**. Primary tumour and metastases in liver and spleen of rats after intraperitoneal injection of MTPa and MTPa‐Tspan8 cells in Fischer rats

**Figure S9**. NTA analysis of blood samples of rats after orthotopic injection of MTPa and MTPa‐Tspan8 cells

**Figure S10**. Nanoparticle tracking analysis (NTA) and dynamic light scattering (DLS) of extracellular vesicles (EVs) isolated from blood of rats after intraperitoneal injection of MTPa and MTPa‐Tspan8 cells

**Figure S11**. Settings for flow cytometry analysis of EVs isolated from the blood of tumour‐bearing animals

**Table S1**. List of the antibodies used (mentioned in supplementary material, Supplementary materials and methods)

**Table S2**. List of the primers used for qPCR (mentioned in supplementary material, Supplementary materials and methods)

**Table S3**. List of the siRNAs used for transient gene knockdown

**Table S4**. Human breast cancer cell lines tested for TSPAN8 expression

**Movie S1**. Time lapse of wound closure by MTPa cells

**Movie S2**. Time lapse of wound closure by MTPa‐Tspan8 cells

## Supporting information


**Supplementary materials and methods**

**Figure S1**. RT‐qPCR analysis of TSPAN8 encoding mRNA in human tumours
**Figure S2**. Impact of Tspan8 on adhesion and proliferation of human breast cancer cells MDA‐MB‐231
**Figure S3**. Customised agarose device to test impact of different ECM on cell growth and behaviour
**Figure S4**. Radiation resistance test of MTPa‐pcDNA3 and MTPa‐Tspan8 cells under 3D conditions
**Figure S5**. Impact of Tspan8 on radiation sensitivity using conventional 2D colony formation assays
**Figure S6**. Co‐regulation of Tspan8 and E‐cadherin expression in MTPa‐Tspan8 cells
**Figure S7**. Co‐regulation of Tspan8 and E‐cadherin expression in MDA‐MB‐361 cells
**Figure S8**. Primary tumour and metastases in liver and spleen of rats after intraperitoneal injection of MTPa and MTPa‐Tspan8 cells in Fischer rats
**Figure S9**. NTA analysis of blood samples of rats after orthotopic injection of MTPa and MTPa‐Tspan8 cells
**Figure S10**. Nanoparticle tracking analysis (NTA) and dynamic light scattering (DLS) of extracellular vesicles (EVs) isolated from blood of rats after intraperitoneal injection of MTPa and MTPa‐Tspan8 cells
**Figure S11**. Settings for flow cytometry analysis of EVs isolated from the blood of tumour‐bearing animals

**Table S1**. List of the antibodies used (mentioned in supplementary material, Supplementary materials and methods)
**Table S2**. List of the primers used for qPCR (mentioned in supplementary material, Supplementary materials and methods)
**Table S3**. List of the siRNAs used for transient gene knockdown
**Table S4**. Human breast cancer cell lines tested for TSPAN8 expression

**Movie S1**. Time lapse of wound closure by MTPa cells

**Movie S2**. Time lapse of wound closure by MTPa‐Tspan8 cells
