# Supplementary material for: Tspan8 is expressed in breast cancer and regulates E‐cadherin/catenin signalling and metastasis accompanied by increased circulating extracellular vesicles
Source: J Pathol. 2019 Jun 18;248(4):421–37. doi: 10.1002/path.5281 (PMC6771825; doi:10.1002/path.5281)
Supplement: Supplementary file 1 — Supplementary materials and methods [file PATH-248-421-s001.docx]

**Tspan8 is expressed in breast cancer and regulates E-cadherin/catenin signalling and metastasis accompanied by increased circulating extracellular vesicles in a rat model**

Voglstaetter M *et al*. *J Pathol* DOI: 10.1002/path.5281

**Supplementary materials and methods**

**Cell lines and cell culture, and generation of the MTPa-Tspan8 sub-line**

The rat cell line MTPa was maintained in DMEM–10% FCS, 2 mm glutamine; all human cell lines applied were obtained from the ATCC. For generation of the MTPa-Tspan8 cell lines, parental MTPa cells were transfected with the pcDNA3.1 plasmid containing full-length cDNA coding for rat Tspan8. As a control, MTPa cells were stably transfected with the pcDNA3.1 plasmid without insert. Stably transfected clones were selected by using G418 1 mg/ml and sorted for the expression of Tspan8. Tspan8 expression was routinely assessed by FACS and sorting was repeated if the proportion of Tspan8^+^ cells was below 65%.

T47D was maintained in RPMI–10% FCS, 2 mm glutamine; MDA-MB-361 was maintained in DMEM–10% FCS, 2 mm glutamine supplemented with pyruvate; MCF7, BT549, MDA-MB-231, and its derivatives were maintained in DMEM:F12 (1:1)–10% FCS, 2 mm glutamine; SUM149 and SUM159 were maintained in F12–5% FCS supplemented with hydrocortisone and insulin.

**AFM-based single-cell force spectroscopy**

Cells were cultured to 20% confluence and transferred into CO_2_-independent medium (Gibco) 1 h prior to the experiment. To prepare probe cells, cells were trypsinised, resuspended in 200 µl of CO_2_-independent medium, and maintained at 37°C. Single-cell force spectroscopy (SCFS) measurements were performed using a CellHesion200 AFM (JPK Instruments) mounted onto an AxioObserver A1 inverted light microscope (Carl Zeiss). For stable cell attachment, plasma-activated tipless cantilevers (NP-O, type D, nominal spring constant 0.06 N/m, Bruker) were functionalised with concanavalin A. After determining the spring constant of the cantilever using the thermal noise method, a single probe cell was immobilised on the cantilever by applying a force of 0.5 nN for 5 s. After a rest period of 5 min, the probe cell was then approached onto a second substrate-attached cell using a contact force of 1.5 nN and contact times between 10 and 300 s, and finally retracted at a speed of 5 µm/s. Between measurements, cells were rested for a period of time corresponding to the contact time used. Usually 4–10 different substrate-immobilised cells were measured per probe cell. Force curves were analysed using JPK Data Processing 4.0 software and the extracted maximum detachment forces were pooled for each cell line and contact time. Statistical analysis was performed using a non-parametric Mann–Whitney test.

**Cell proliferation, adhesion, cluster formation, and migration**

To measure cell proliferation, the cells were incubated for 24–96 h in the complete medium. [^3^H]Thymidine was added during the last 16 h; incorporation was measured in a β-counter.

For cell adhesion, cells were seeded in pre-coated 24-well plates. After 1 h, cells were gently rinsed with PBS, fixed, and stained with 0.1% crystal violet solution. After treatment with 10% acetic acid, 595 nm optical density was measured in a microplate reader (Tecan).

Time-lapse microscopy was used to examine cluster formation and migration. For cluster formation, cells were seeded in 12-well plates and documented using Axiovert 200M Life Imaging Systems (Carl Zeiss) equipped with a heated chamber supplemented with 5% CO_2_. Images were taken every 15 min for 10 h. Migration was assessed using a conventional wound healing assay. Images were taken every 20 min for 24 h. For calculation, ImageJ Plugin was used, allowing quantification of cell-free areas.

**2D colony formation and irradiation resistance assay**

For 2D colony formation, triplicates 100 cells per dish were seeded in 10 cm dishes, cultured for 10 days, fixed with 3% formalin, stained with crystal violet, and counted. Statistical analysis was performed using a two-tailed *t*-test.

To test irradiation resistance 1000–5000 cells per 10 cm dish were seeded and gamma-irradiated using ^137^Cs at 0.66 Gy/min on day 2 for the desired irradiation dose. After irradiation, cells were maintained in culture for 2–3 weeks until visible colonies were formed. The number of cell colonies was counted as described above. Statistical analysis was performed using a two-tailed *t*-test.

**3D colony formation, proliferation, and irradiation resistance assay**

For 3D colony-forming ability, single cell suspension in 0.3% agar was loaded on a 0.5% agar cushion. Visible colonies were counted after 14 days using 0.005% crystal violet solution for staining. Statistical analysis was performed using two-tailed *t*-tests.

3D growth was tested in recently developed microwell arrays (abc biopply AG, Solothurn, Switzerland). Cells were seeded 25 000 cells/cm² and maintained for 10 days unless otherwise mentioned. MTT staining was performed at day 10; the aggregates in agarose inserts were counted by optical scanning on a high-resolution flatbed scanner CanoScan 9000F MarkII (Canon Inc) and analysed using ImageJ (NIH).

To test irradiation resistance 10 cells per well were seeded into the microwell arrays and gamma-irradiated using ^137^Cs at 0.66 Gy/min on day 2 for the desired irradiation dose. After irradiation, cells were maintained in culture for another 10 days and the number of cell aggregates was then counted as described above.

**RNA isolation and RT-qPCR**

Cellular mRNA was extracted using an RNeasy Mini kit (Qiagen); human RNA was isolated using TRIzol (Invitrogen); 4 µg of total RNA was treated with 10 U of DNase I prior to reverse transcription and cDNA production. Twenty-five nanograms of cDNA was amplified with SYBR^®^ Green (Qiagen) using gene-specific primers (supplementary material, Table S2) and analysed using an ABI PRISM 7000 (Applied Biosystems). *Rib2* and *GAPDH* were used as controls. Statistical analysis was performed using the Δ*C*_t_ method according to the supplier’s recommendations.

**RNA silencing**

For transient knockdown, 20 pmol gene-specific siRNAs (FlexiTube, Qiagen) and controls (Table S3): AllStars-AlexaFluor488, siRNAsScramble (Qiagen) were transfected using ScreenFect (Incella). The knockdown was confirmed using RT-qPCR, WB, and FACS after 24, 48, and 72 h if not otherwise mentioned. siRNA experiments were performed in technical duplicates and biological triplicates.

**Western blotting (WB) and immunoprecipitation**

Cells were washed in HEPES buffer (25 mm HEPES, pH 7.4, 150 mm NaCl, 5 mm MgCl_2_) and where required, incubated with 5 mm water-soluble BS^3^ reagent (Pierce) (1 h, 4°C). If EVs were used for immunoprecipitation, EVs were diluted in PBS, and the protein concentration and EV number were measured prior to EV lysis. For each immunoprecipitation, 2 µg of EV proteins were used. Cells and EVs were lysed [HEPES buffer, 1% CHAPS (for IP) or Triton X-100 (for WB), 1 mm phenylmethylsulphonyl fluoride, EDTA-free protease inhibitor mix] for 45 min at 4°C. For immunoprecipitation, lysates were incubated with the indicated antibody (2 h) and protein G Sepharose (1 h). After washing (four times), lysates/immunoprecipitates were resolved on 12% non-reducing SDS-PAGE and subjected to immunoblotting using HRP-conjugated antibody following enhanced chemiluminescence-based signal detection with SuperSignal West DURA Extended Duration Substrate (Pierce).

All antibodies are listed in the supplementary material, Table S1.

**Immunohistochemistry**

We obtained the tissue for our examination from paraffin-embedded tissue blocks, which were cut into 2 µm sections and placed on SuperFrost Plus adhesion slides. We used an oven to melt excess paraffin at 70°C in a moist atmosphere for 20 min. Sections were dewaxed and rehydrated using two baths of xylol (100%) and then a series of decreasing concentrations of ethanol (100%, 96%, 90%, 80%, 70%, and 70%) for 5 min each bath. Citrate buffer was pre-heated in a steam cooker and sections were incubated in approximately 90°C hot citrate buffer for 20 min. The slides were washed in TBS buffer solution and then blocking solution from the DAKO Real-Kit for 30 min with the slides in a moist atmosphere. We used a 1:50 concentration of the primary antibody TS29 against Tspan8 and incubated it overnight. The next day, the primary antibody was removed and the secondary antibody in the DAKO Real-Kit was incubated for 60 min and the HRP-link from the same kit for 30 min. Chromogen substrate was applied for 20 s and the staining reaction was controlled by light microscopy. Finally, we covered the slides with a 1:1 dilution of Vitro-Clud^®^ and xylol as a mountant.

**Immunofluorescence of frozen sections**

Primary tumours and organs were snap-frozen in liquid nitrogen and stored at –80°C prior to preparing cryosections. Seven-micrometre-thick cryosections were prepared and stored at –80°C. Prior to staining, the sections were fixed in ice-cold 4% paraformaldehyde, washed with 1× PBS, and blocked with 1% horse serum for 1 h. Then sections were incubated in a humidified chamber with Tspan8 antibody (2% horse serum in PBS, 0.05% Triton X-100) for 1 h, room temperature, washed, incubated with AlexaFluor-594 secondary antibody for 1 h, and then with DAPI solution for 10 min. Sections were washed, dried, and mounted with DAKO anti-fade mounting medium. Images were taken using a Leica SP2 AOBS confocal microscope.

**Isolation of EVs from the cell culture supernatant**

For EV preparation, 300 ml cell culture supernatants were clarified at 2000 × *g*, 20 min; 9000 × *g*, 45 min at 4°C; filtered through a 0.22 µm filter to remove larger particles; and pelleted by ultracentrifugation at 120 000 × *g* for 90 min at 4°C. EV pellets were washed and resuspended in 1× PBS. All EV preparations were routinely assessed by NTA, EM, and expression of exosome markers: CD9, CD81, and Alix.

**Isolation of EVs from rat serum**

After intracardiac blood collection, samples were transferred into serum microtubes 1.1 ml Z-Gel (Sarstedt) and centrifuged at 3000 × *g* at 4°C for 10 min to collect rat serum. For EV isolation, 400 μl of rat serum was diluted and centrifuged at 4600 × *g*, 4°C for 30 min and at 12 000 × *g*, 4°C for 45 min to remove cell debris and larger vesicles. EVs were precipitated with 8% PEG for 60 min at 4°C after centrifugation at 1500 × *g*, 4°C for 30 min. The resulting pellet was resuspended to 250 μl using double-distilled H_2_O (ddH_2_O) and subjected to the follow-up analyses: NTA, DLS, and western blotting.

**Electron microscopy**

All preparations of extracellular vesicles were characterised by transmission electron microscopy (TEM). Ten microlitres of each vesicle preparation was loaded on a 300-mesh copper grid and fixed with 1% glutaraldehyde, washed with ddH_2_O, and negatively stained by applying a 10 µl drop of 1% uranyl acetate. The images were taking using an electron microscope (LEO 906 E; Zeiss, Oberkochen, Germany) using SIS software (Olympus, Hamburg, Germany).

**Nanoparticle tracking analysis (NTA)**

Samples were diluted consecutively with ddH_2_O to receive an optimal concentration of 100–200 particle counts. One millilitre of diluted sample was injected into the NTA. Size distribution was measured in scatter mode and Zeta potential was measured in Zeta potential mode using the set-up: sensitivity 85; min size 20 nm; max size 1000 nm; min brightness 10.

**Dynamic light scattering**

Twenty microlitres of undiluted sample was placed on the measuring probe. Each sample was measured five times for 60 s and the results were averaged. Results were transferred from intensity mode to volume mode. Set-up was as follows: refractive index 1.33; min size 0.8 nm; max size 6540 nm; viscosity: low temperature 1.002; high temperature 0.797; transparency ‘on’; shape: spherical.
