# Supplementary material for: Tspan8 is expressed in breast cancer and regulates E‐cadherin/catenin signalling and metastasis accompanied by increased circulating extracellular vesicles
Source: J Pathol. 2019 Jun 18;248(4):421–37. doi: 10.1002/path.5281 (PMC6771825; doi:10.1002/path.5281)
Supplement: Supplementary file 2 — Figure S1. RT‐qPCR analysis of TSPAN8 encoding mRNA in human tumours Figure S2. Impact of Tspan8 on adhesion and proliferation of human breast cancer cells MDA‐MB‐231 Figure S3. Customised agarose device to test impact of different ECM on cell growth and behaviour Figure S4. Radiation resistance test of MTPa‐pcDNA3 and MTPa‐Tspan8 cells under 3D conditions Figure S5. Impact of Tspan8 on radiation sensitivity using conventional 2D colony formation assays Figure S6. Co‐regulation of Tspan8 and E‐cadherin expression in MTPa‐Tspan8 cells Figure S7. Co‐regulation of Tspan8 and E‐cadherin expression in MDA‐MB‐361 cells Figure S8. Primary tumour and metastases in liver and spleen of rats after intraperitoneal injection of MTPa and MTPa‐Tspan8 cells in Fischer rats Figure S9. NTA analysis of blood samples of rats after orthotopic injection of MTPa and MTPa‐Tspan8 cells Figure S10. Nanoparticle tracking analysis (NTA) and dynamic light scattering (DLS) of extracellular vesicles (EVs) isolated from blood of rats after intraperitoneal injection of MTPa and MTPa‐Tspan8 cells Figure S11. Settings for flow cytometry analysis of EVs isolated from the blood of tumour‐bearing animals [file PATH-248-421-s002.pdf]

Supplementary Figure S1

RT-qPCR analysis of Tspan8 - coding mRNA in human tumours

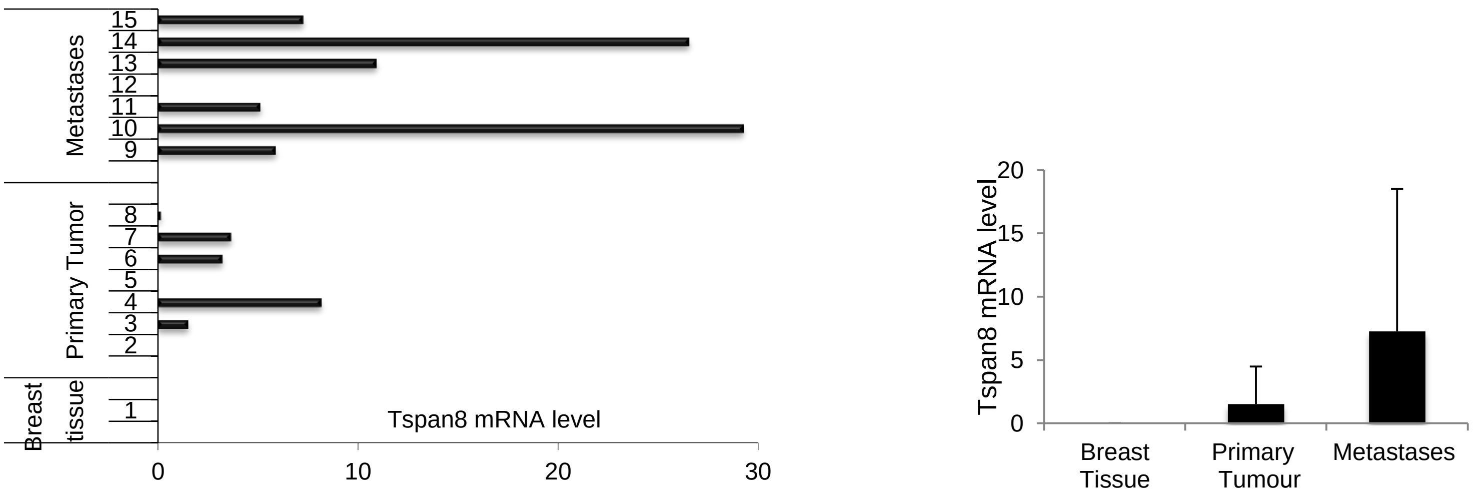

Tspan8 mRNA expression was tested by RT-qPCR in 15 samples: 7 biopsies of breast cancer patients, 7 lung and lymph node metastases and 1 tumour-free sample as a control. The data show a heterogeneous expression of Tspan8 mRNA in primary tumours and metastases with a higher expression range and higher heterogeneity in metastases as compared to the primary tumours.

Supplementary Figure S2

Impact of Tspan8 on adhesion and proliferation of human breast cancer cells MDA-MB-231

A

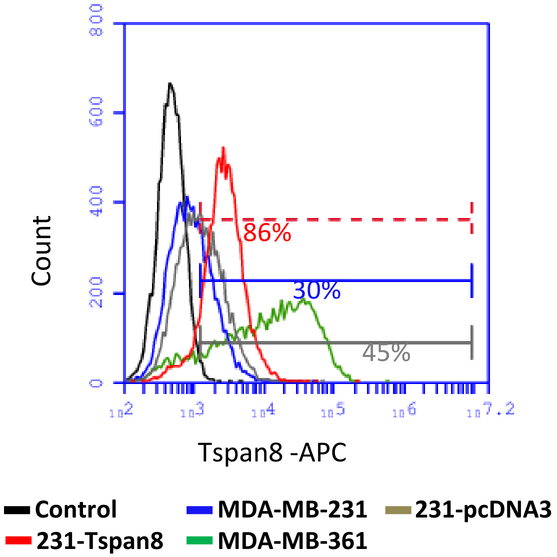

B

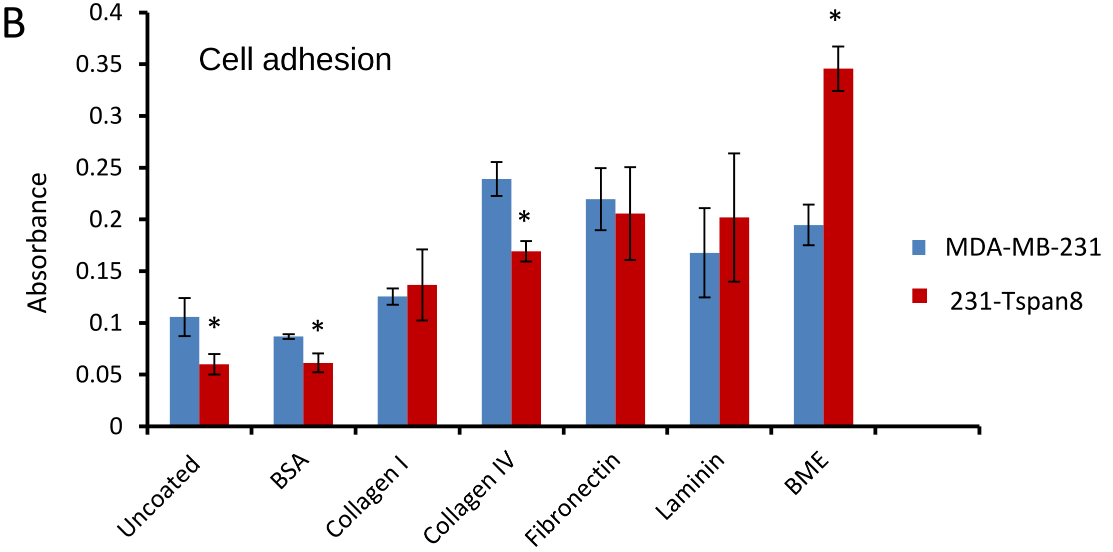

C

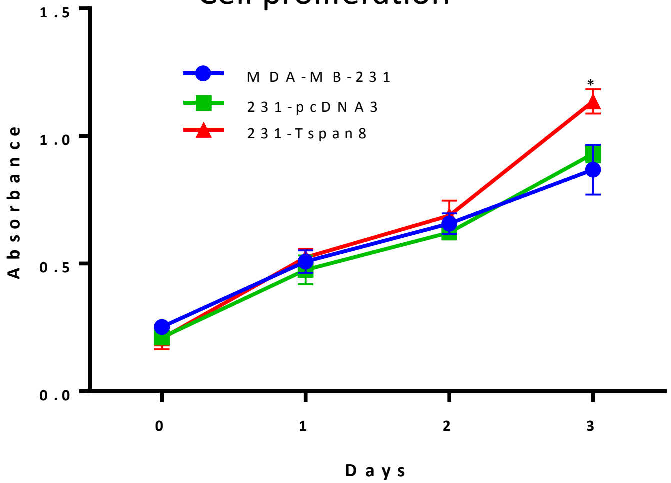

MDA-MB-231 cells were stably transfected with pcDNA3.1 containing full length Tspan8 cDNA. Cells transfected with pcDNA3.1 plasmid without insert were used as a control.

A) FACS was used to assess Tspan8 surface expression in transfected and parental cells. As a positive control MDA-MB-361 cells were used.

B) cell-matrix adhesion was tested using different extracellular matrices (ECM). Cells were seeded on different matrices and analyzed after 1 h. While significantly lower adhesion of the 231-Tspan8 cells was observed on the uncoated surfaces, BSA control and Collagen IV, they showed a significantly stronger adhesion on BME.

C) To test impact of Tspan8 on cell proliferation, WST1 test, showing metabolic activity of cells, was used. Cells were seeded in 96-well plates and monitored for 3 d. Untransfected MDA-MB-231 and 231-pcDNA3 were used as a control. The 231-Tspan8 cells exhibited significantly higher proliferation compared to the controls. All experiments were repeated 3 times in quadruplicates. Expression of Tspan8 in cells was assessed every 2 months.

Supplementary Figure S3
Customised agarose device to test impact of different ECM on cell growth and behaviour

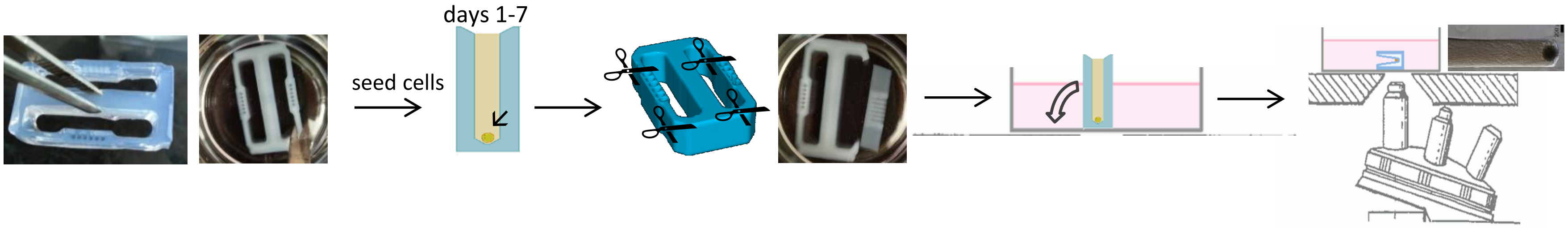

1000 cells were seeded into sterile agarose cavities and maintained for 1 day in medium to form aggregates.
Cavities were then filled with a desired extracellular matrix (collagen I or BME) and maintained in culture for 7 d.
For imaging, agarose bars were disconnected from the carrier and flipped 90°.
Images were taken using bright-field microscopy, then quantitative analysis was performed using ImageJ.

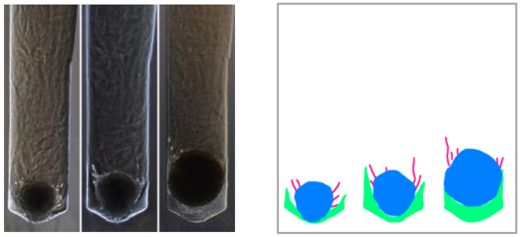

d2 d4 d7 d2 d4 d7

For quantification, images were taken using 4x objective and a resolution of 612 pixel / 1000 µm.
In each image, contours of cell aggregate (blue) were marked manually using ImageJ and quantified. For each group at least 8 cell aggregates were evaluated (range: 8 – 15). For each cell line the experiment was performed at least twice.
Using ImageJ the area of cell aggregates was calculated in µm². Additionally, length of outgrowths was assessed.

Supplementary Figure S4
Radiation resistance test of MTPa-pcDNA3 and MTPa-Tspan8 cells under 3D conditions

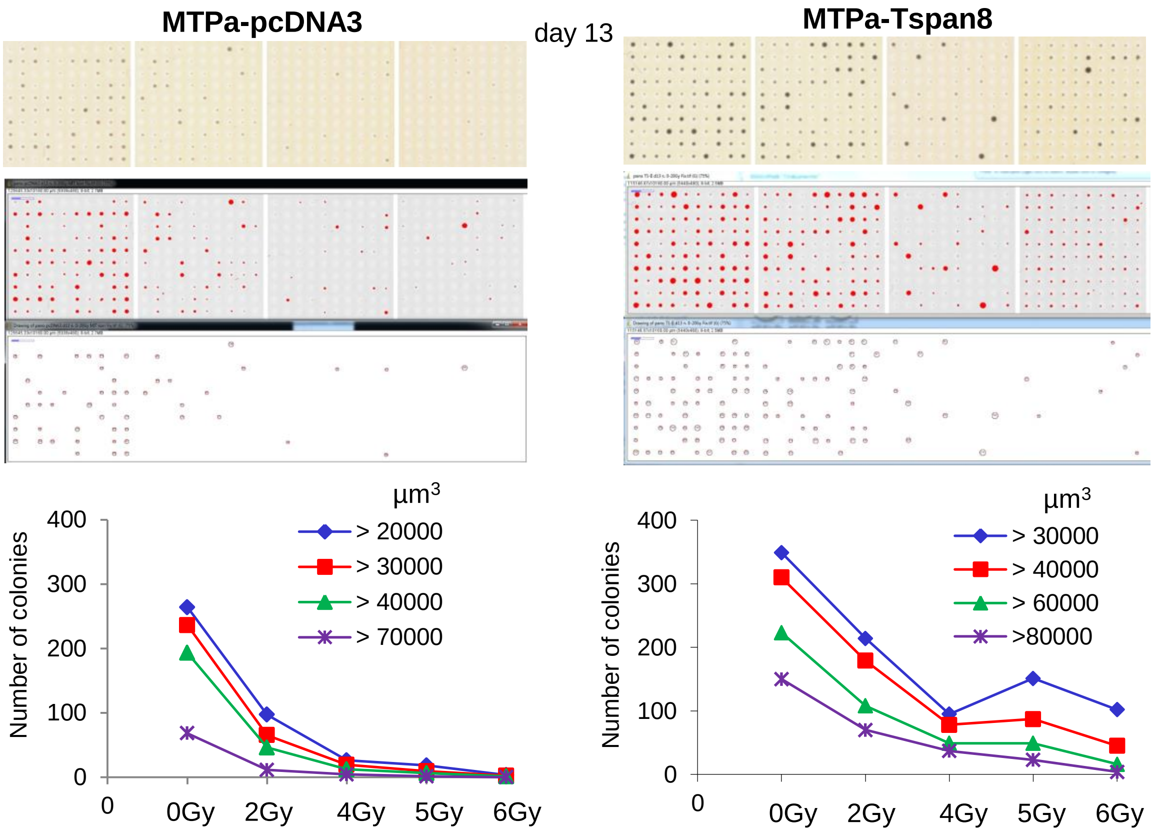

To assess radiation resistance, anchorage independent colony formation ability of cells upon ionizing irradiation was tested. For that, 10 cells/microwell were seeded in customised agarose inserts with 100 microwells/cm^2. On day 2, cells were exposed to different radiation doses using 137 Cs at 0.66 Gy/min and maintained for 13 d.

To enhance visibility, MTT (3-(4,5-dimethylthiazol-2-yl)-2,5-diphenyltetrazoliumbromide) staining was performed on day 13. Agarose inserts were scanned on a high-resolution flatbed scanner (CanoScan 9000F Mark II, Canon Inc.), using the transmitted light modus at 1200 dpi. The projected areas of individual cell aggregates was measured using ImageJ.

Tspan8 strongly enhanced cellular resistance to irradiation. A fraction of MTPa-Tspan8 cells maintained colony formation ability following 6 Gy, whereas the number of colonies built by the parental cells was already reduced by more than 50 % after treatment with 2 Gy. It was noted that multicellular aggregates exhibited a variable resistance, resembling the variations in metastases response observed after radiotherapy of patients in the clinical setting.

Supplementary Figure S5

Impact of Tspan8 on radiation sensitivity using conventional 2D colony formation assays

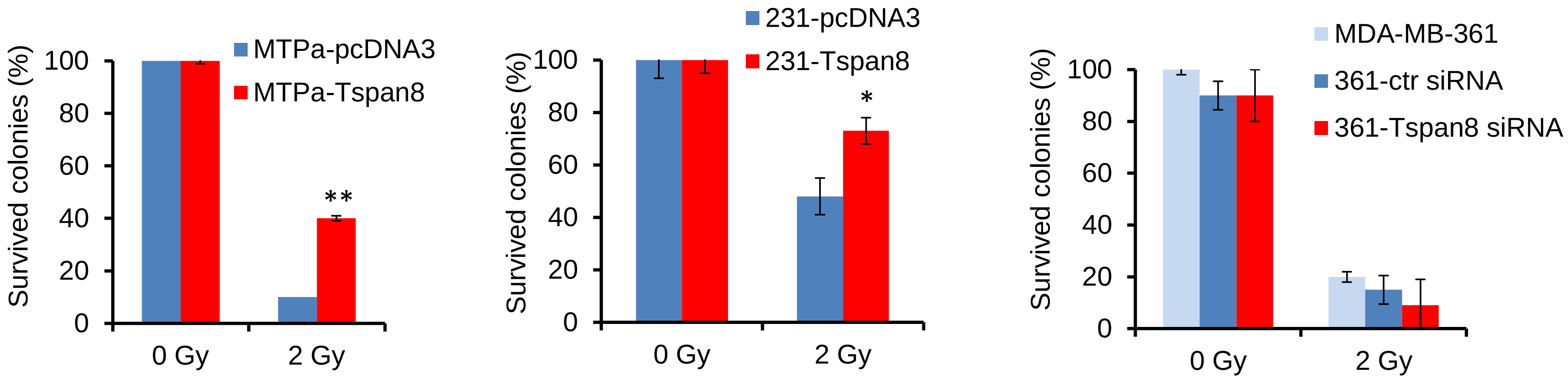

To test radiation sensitivity of different cell lines, colony forming assays were used as described elsewhere [30, 31]. Cells were seeded at 1000–5000 cells / 10 cm plate and irradiated at 2 Gy, established as optimal radiation power to test breast cells radiosensitivity [31]. For siRNA, cells were transfected in 24-well plates and transferred after 1 day into 10 cm dishes. Survival data were corrected for siRNA toxicity as described [31]. After 14 d, the cells were fixed and stained with crystal violet. Images were taken and visible colonies were counted using ImageJ.

Tspan8 showed a significant effect on radiation resistance in MTPa and MDA-MB-231 cells. Knockdown of Tspan8 in MDA-MB-361 cells resulted in a slight but not significant decrease of radiation resistance,

Supplementary Figure S6
Co-regulation of Tspan8 and E-cadherin expression in MTPa-Tspan8 cells

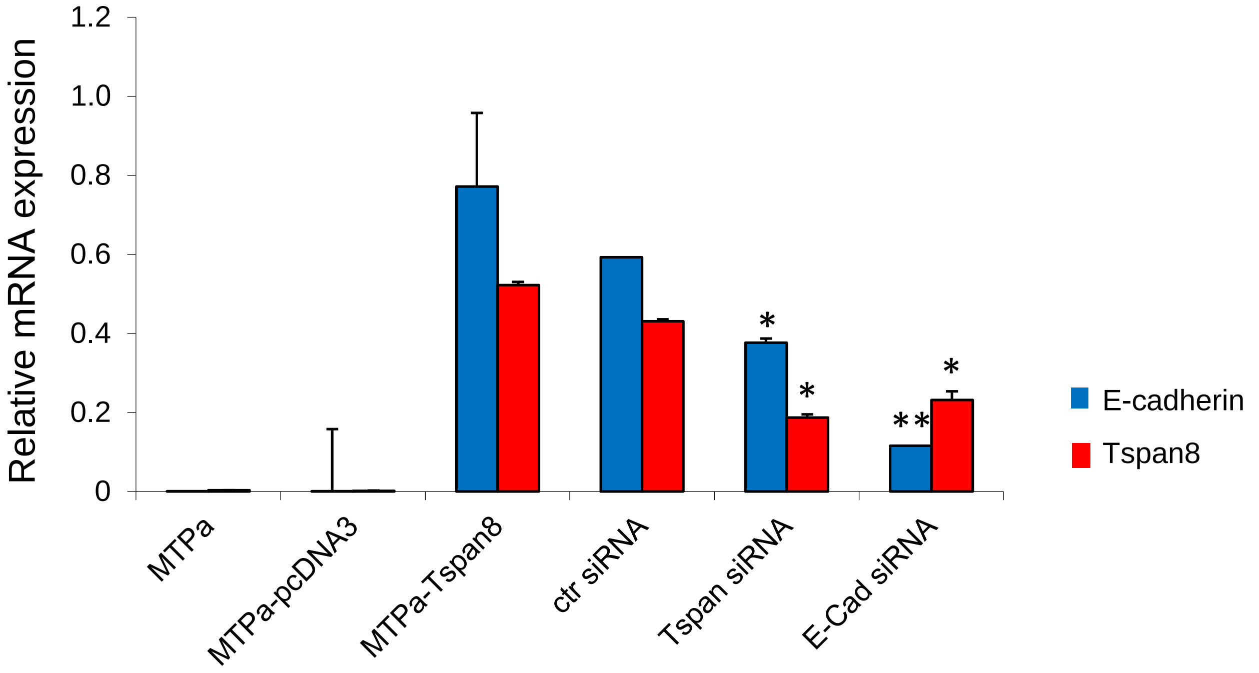

To test co-regulation of E-Cadherin and Tspan8, MTPa-Tspan8 cells were transfected with Tspan8-siRNA and E-Cadherin-siRNA. As controls MTPa, MTPa-pcDNA3, MTPa-Tspan8 untreated and MTPa-Tspan8 cells transfected with scrambled siRNA were used. RNA was harvested 48 h post-transfection and RT-qPCR was performed. Significant downregulation of Tspan8 mRNA was achieved by both Tspan8-siRNA and E-cCadherin-siRNA. Conversely, significant E-cadherin knockdown was achieved by E-cadherin and Tspan8-siRNA, indicating their co-regulation..

Supplementary Figure S7
Co-regulation of Tspan8 and E-cadherin expression in MDA-MB-361 cells

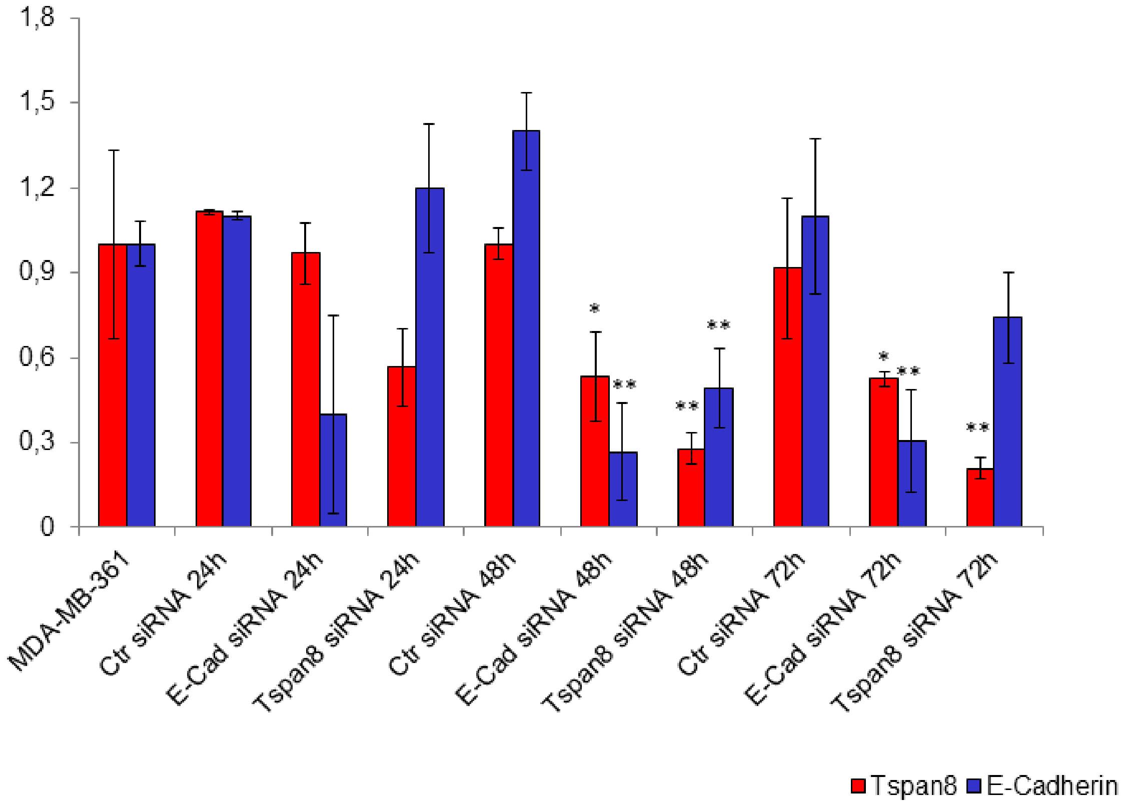

Co-regulation of E-cadherin and Tspan8 encoding mRNAs was examined in human MDA-MB-361 cells by RT-qPCR. MDA-MB-361 cells expressing endogenous Tspan8 and E-cadherin were transfected with the corresponding siRNAs and analysed after 24, 48 and 72 h. No significant regulation was detected after 24 h. After 48 h, a knockdown of Tspan8 by siRNA led to a significant down-regulation of both, Tspan8 and E-cadherin mRNA, and conversely knockdown of E-Cadherin by siRNA resulted in a significant down-regulation of Tspan8 and E-Cadherin mRNAs, indicating their co-regulation. Significance was calculated according to the mRNA expression levels in cells transfected with scrambled siRNA after 48 h (Ctr siRNA 48 h). After 72 h, both, Tspan8 and E-Cadherin mRNAs were significantly downregulated in the cells transfected with E-cadherin siRNA; only Tspan8 mRNA was downregulated in the cells transfected with Tspan8 siRNA. Significance was calculated according to the mRNA expression level following transfection with the scrambled siRNA after 72 h (Ctr siRNA 72 h).

Supplementary Figure S8

Primary tumour and metastases in liver and spleen of rats after intraperitoneal injection of MTPa and MTPa-Tspan8 cells in Fischer rats

A

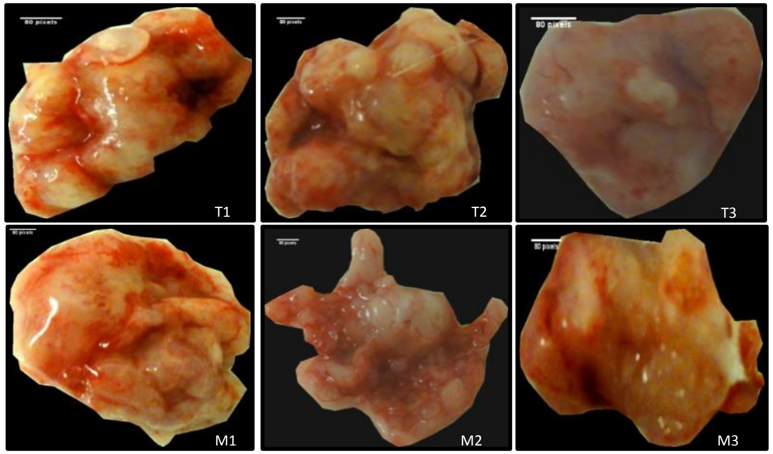

B

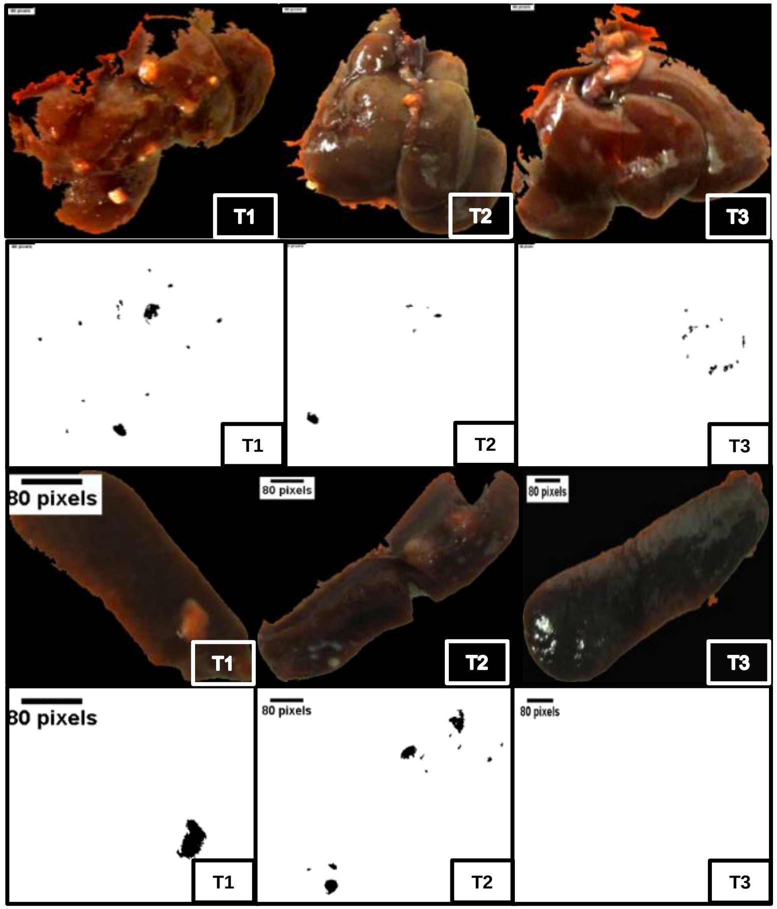

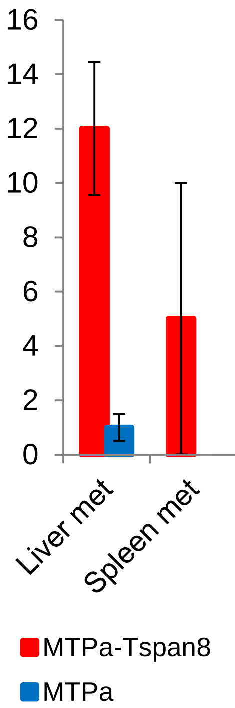

Rats were injected with 1 million MTPa or MTPa-Tspan8 cells. 3 wk after injection, the animals were sacrificed and primary tumours and organs with metastases were isolated.

(A) Images of primary tumours

T1, T2, T3 - primary tumour of animals injected with MTPa-Tspan8 cells

M1, M2, M2 – primary tumour of animals injected with MTPa cells

(B) Counting of metastases in liver and spleen

Each image was processed to an 8 bit image and the background was subtracted. To determine metastases on liver and spleen surface from healthy tissue, a suitable threshold was set by using Auto Threshold. Set ups for counting metastases were size: 20-2000 pixels and circularity: 0.1– 1.00. Brightness and contrast of images were adjusted and a scale bar was included. All image editing was performed with ImageJ.

Supplementary Figure S9
NTA Analysis of blood samples of rats after orthotopic injection of MTPa and MTPa-Tspan8 cells

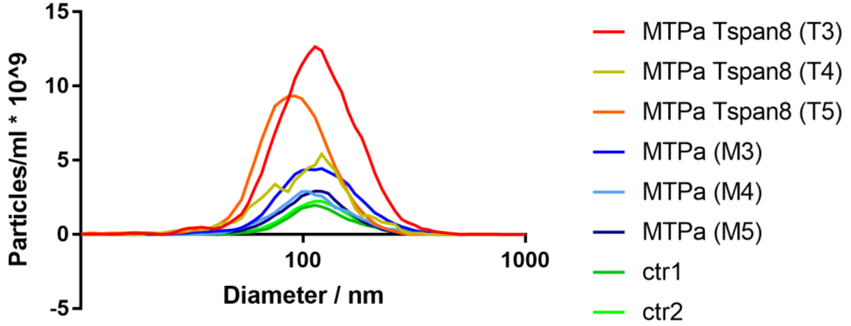

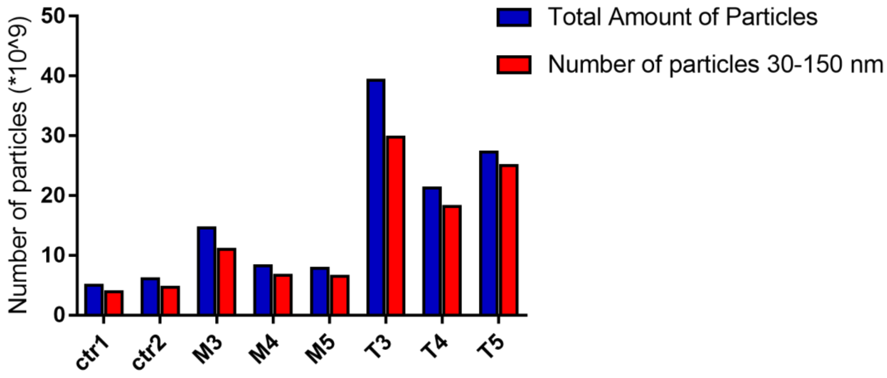

1 x 10^6 MTPa or MTPa-Tspan8 cells were injected orthotopically into a mammary fat pad of rats (5 animals / group). Blood was collected from each rat at day 18 and EVs were isolated by a customised filtration / precipitation method, allowing for a high EV yield; 2 control samples, 3 samples from rats after MTPa cell injection and 3 samples from rats after MTPa-Tspan8 injection were assessed using NTA. Here, single measurements are shown. Left panel- NTA size distribution. Right panel – calculation of particle number/ 1 ml of serum.

Significantly more EVs were detected in the blood of each of the rats tested that had been injected with MTPa-Tspan8 cells.

Supplementary Figure S10

Nanoparticle Tracking Analysis (NTA) and Dynamic Light Scattering (DLS) of Extracellular Vesicles (EV) isolated from blood of rats after intraperitoneal injection of MTPa and MTPa-Tspan8 cells

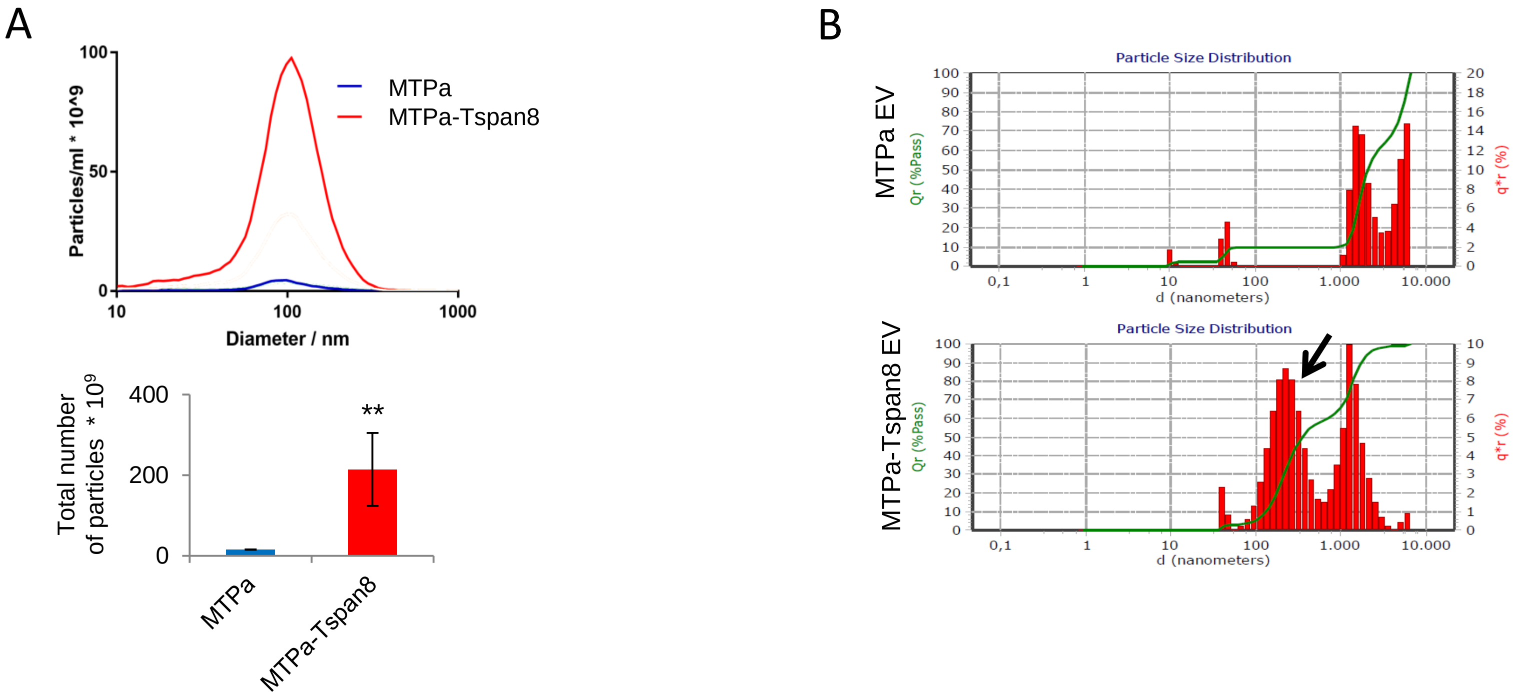

EVs were isolated using a customised filtration / precipitation method from 200 µl of rat serum taken 3 weeks after intraperitoneal injection and analysed using nanoparticle tracking analysis (NTA) (A) and dynamic light scattering (DLS) (B), the latter, is the only technique allowing detection of a wide range of particles from 3 nm to 10 µm.

A) No difference in size distribution between EVs derived from the MTPa and MTPa-Tspan8 injected animals were observed as shown by the size distribution diagrams (upper panel). However, a several-fold greater number of EVs was detected in animals harbouring the MTPa-Tspan8 tumour (bottom panel).

B) DLS revealed that serum of rats injected with MTPa-Tspan8 cells contained a population of vesicles 100–1000 nm (black arrow) not detectable by DLS in the rats injected with MTPa cells. Taking into account the detection limit of the DLS device, the result indicates that the EVs of size (100–1000 nm) may be present in the serum derived from the MTPa-injected rats at a significantly lower level. Each animal was tested and representative diagrams are shown.

Supplementary Figure S11

Settings for flow cytometry analysis of EVs isolated from the blood of tumour-bearing animals

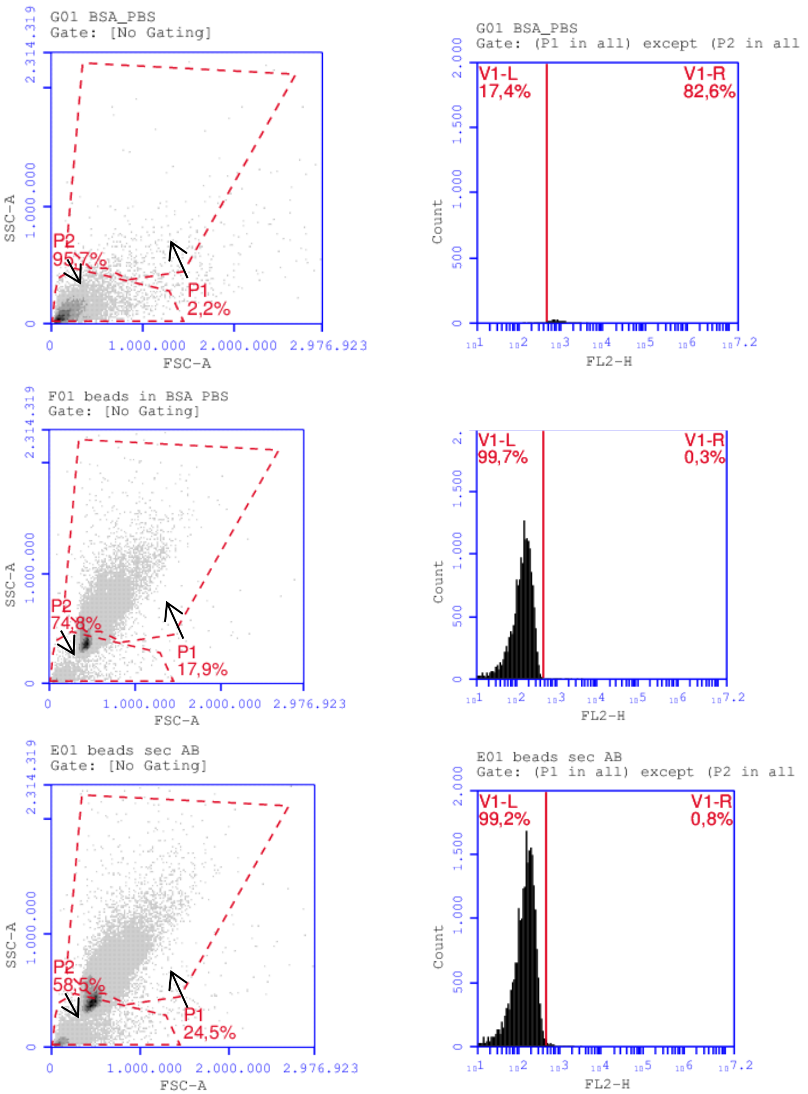

3%BSA/1xPBS without beads

Latex beads in 3%BSA/1xPBS

Latex beads with isoptype control (mouse IgG 1:100 in 3%BSA/1xPBS)
and secondary AB (anti mouse-PE, 1:50 in 1%BSA/1xPBS)

To perform flow cytometry of EVs isolated from the blood of tumour-bearing animals, the EVs were bound on 4 µm diameter latex beads. To establish settings of the device (gates P1 and P2), buffer without beads (upper panel), beads in BSA / PBS and beads in BSA /PBS containing latex beads and beads bound with EVs, isotype control and secondary antibody were measured in a flow cytometer. The P2 gate was excluded from the analysis, because it contained a majority of unspecific contaminants. P1 contained a signal corresponding to beads and beads with EVs.
