# Supplementary material for: Tspan8 is expressed in breast cancer and regulates E‐cadherin/catenin signalling and metastasis accompanied by increased circulating extracellular vesicles
Source: J Pathol. 2019 Jun 18;248(4):421–37. doi: 10.1002/path.5281 (PMC6771825; doi:10.1002/path.5281)
Supplement: Supplementary file 3 — Table S1. List of the antibodies used (mentioned in supplementary material, Supplementary materials and methods) Table S2. List of the primers used for qPCR (mentioned in supplementary material, Supplementary materials and methods) Table S3. List of the siRNAs used for transient gene knockdown Table S4. Human breast cancer cell lines tested for TSPAN8 expression [file PATH-248-421-s003.docx]

**Tspan8 is expressed in breast cancer and regulates E-cadherin/catenin signalling and metastasis accompanied by increased circulating extracellular vesicles in a rat model**

Voglstaetter M *et al*. *J Pathol* DOI: 10.1002/path.5281

**Table S1.** List of the antibodies used

| **Antibody** | **Supplier** | **Used dilution** |
| --- | --- | --- |
| **Primary antibodies** |  | |
| D6.1, hybridoma | Kindly provided by M Zöller, Experimental Surgery, University Clinic, Heidelberg* | |
| Tspan8 (TS29) | Produced by Boucheix group as described elsewhere [14] | |
| Tspan8 (MAB4734) | R&D Systems, Heidelberg, Germany | 1:200 |
| Actin (mABGEa) | Thermo Fisher Scientific, Mainz, Germany | 1:2000 |
| Beta-catenin (E-5)) | Santa Cruz, Heidelberg, Germany | 1:500 |
| Cadherin-11 (16G5) | BioLegend, San Diego, CA, USA | 1:200 |
| CD9 (H-110) | Santa Cruz | 1:500 |
| GAPDH (G-9)) | Santa Cruz | 1:500 |
| E-Cadherin (DECMA-1) | BioLegend | 1:800 |
| p120-catenin (H-90) | Santa Cruz | 1:200 |
| TSG101 (C-2) | Santa Cruz | 1:200 |
| Tubulin (DLN-15303) | Dianova, Hamburg, Germany | 1:500 |
| Vinculin (H-10) | Santa Cruz | 1:500 |
| CD9 (M-L13) | BD Biosciences, Allschwil, Switzerland | 1:1000 |
| **Secondary antibodies** |  | |
| Goat anti-mouse, HRP-conjugated | Dako, Jena, Germany | 1:1000 |
| Goat anti-rabbit, HRP-conjugated | Dako | 1:1000 |
| Goat anti-mouse, Alexa-488 conjugated - | Thermo Fisher Scientific | 1:500 |
| Goat anti-rabbit, Alexa-594 conjugated - | Thermo Fisher Scientific | 1:500 |

*EAACC: European Association of Animal Cell Cultures, Porton Down, UK.

**Table S2.** List of the primers used for qPCR

| Rat | rTwist fw | CTCGGCAGGCCGGAGACCTA |
| --- | --- | --- |
| Rat | rTwist rv | TCCACCGGCCTGTCTCGCTT |
| Rat | rCdh1 fw | ACCGGCCCAGGAGCTGAC AA |
| Rat | rCdh1 rv | GGATCCTCCACG GCTTCCCCA |
| Rat | rTM4SF3 fw | TGGGATGCTGCGGAGCTGTG |
| Rat | rTM4SF3 rv | AGCTCCTAGGATACCTGCGGC |
| Rat | rCTNNB1 fw | AGCAACAAGCCGGCCATCGT |
| Rat | rCTNNB1 rv | GCTGCGCAGGTGACCACATT |
| Rat | rCTNND1 fw | TGGACCATGCGCTACACGCC |
| Rat | rCTNND1 rv | CCGAAGTTTCCGCCGGGCTT |
| Rat | rLEF1 fw | ATCCCATCACGGGTGGGTTC |
| Rat | rLEF1 rv | GCTGGATGAGGGATGCCAGT |
| Rat | rAxin2 fw | GCGAGCGTGAGATCCACAGA |
| Rat | rAxin2 rv | CATGGAATCGTCGGTCAGCG |
| Rat | rNKD1 fw | ACCCCGACAGCTGAGATTGG |
| Rat | rNKD1 rv | CGGAAGGGCCACTTCTAGGG |
| Rat | rNKD2 fw | ATCATTCCTCGGGGAGCAGC |
| Rat | rNKD2 rv | CTGGGGATCTGTGTTGGGCT |
| Rat/human | Rib_rv | CCGGATATGAGGCAGCAG |
| Rat/human | Rib_fw | GAAGGCTGTGGTGCTGATGG |
| Human | hGAPDH-fw | GGCAAATTCCATGGCACCGT |
| Human | hGAPDH-rv | GCAAATGAGCCCCAGCCTTC |
| Human | hTM4FS3-fw | GGCTTCCTGGGATGCTGCGG |
| Human | hTM4SF3-rv | TCCCCTGTGGCGCTCAAAAGC |
| Human | hCDH1-fw | TCCGGACACTGGTGCCATTT |
| Human | hCDH1-rv | AGGGCTGTGTACGTGCTGTT |
| Mouse | mTm4sf3-fw | GTTCAAGTGCTGTGGCTTGG |
| Mouse | mTm4sf3-rv | GGGTAAACGGAACTCCCCTG |

**Table S3.** List of the siRNAs used for transient gene knockdown

| **Target gene** | **Target sequence** | **Supplier** |
| --- | --- | --- |
| *TM4SF3* | TAGCAATATGGGTACGAGTAA | Qiagen, Hilden, Germany |
| *CDH1* | CAGCGACTGGTTCAGATCAAA | Qiagen |
|  | **Sense sequence** |  |
| scrmlRNA1 – canine survivin | UACGAUCGAAAGCAAAGAAAAGACG | Qiagen |
| scrmlRNA2 – canine survivin | CGUCUUUUCUUUGCUUUCGAUCGUA | Qiagen |
| Transfection control | All Stars Neg, siRNA AF488, Cat. Number 1027284 * | Qiagen |

*No sequence was obtained for the All Stars negative control siRNA from the supplier.

**Table S4.** Human breast cancer cell lines tested for TSPAN8 expression

| **Cell line** | **Origin** | **E-cadherin expression** | **TSPAN8 expression** | **Molecular subtype** |
| --- | --- | --- | --- | --- |
| **BT474** | Invasive ductal carcinoma, primary tumour | + | +/low | Luminal B |
| **BT549** | Invasive ductal carcinoma, primary tumour | − | − | Triple negative |
| **MDA-MB-231** | Adenocarcinoma, metastatic sites, pleural effusion |  | −/low | Triple negative |
| **MDA-MB-361** | Adenocarcinoma, brain metastases | ++ | ++ | Luminal B |
| **MCF7** | Invasive ductal carcinoma, metastatic sites, pleural effusion | + | − | Luminal A |
| **SUM149** | Inflammatory ductal carcinoma | − | − | Triple negative |
| **SUM159** | Anaplastic carcinoma | − | − | Triple negative |
| **T47D** | Invasive ductal carcinoma, primary tumour | + | − | Luminal A |

*All cell lines were purchased from the American Type Culture Collection (Manassas, VA, USA).
